# Population structure analysis and laboratory monitoring of *Shigella* by core-genome multilocus sequence typing

**DOI:** 10.1038/s41467-022-28121-1

**Published:** 2022-01-27

**Authors:** Iman Yassine, Sophie Lefèvre, Elisabeth E. Hansen, Corinne Ruckly, Isabelle Carle, Monique Lejay-Collin, Laëtitia Fabre, Rayane Rafei, Dominique Clermont, Maria Pardos de la Gandara, Fouad Dabboussi, Nicholas R. Thomson, François-Xavier Weill

**Affiliations:** 1 Institut Pasteur, Université de Paris, Unité des bactéries pathogènes entériques, Centre National de Référence des Escherichia coli, Shigella et Salmonella, Paris, F-75015 France; 2 Laboratoire Microbiologie Santé et Environnement (LMSE), Doctoral School of Sciences and Technology, Faculty of Public Health, Lebanese University, Tripoli, Lebanon; 3 Institut Pasteur, Université de Paris, Collection de l’Institut Pasteur, F-75015 Paris, France; 4 Wellcome Sanger Institute, Cambridge, CB10 1SA UK; 5 London School of Hygiene and Tropical Medicine, London, WC1E 7HT UK; 6 Present Address: Harvard Medical School, Boston, MA 02115 USA

**Keywords:** Bacterial genomics, Policy and public health in microbiology, Phylogenomics, Genome informatics

## Abstract

The laboratory surveillance of bacillary dysentery is based on a standardised *Shigella* typing scheme that classifies *Shigella* strains into four serogroups and more than 50 serotypes on the basis of biochemical tests and lipopolysaccharide O-antigen serotyping. Real-time genomic surveillance of *Shigella* infections has been implemented in several countries, but without the use of a standardised typing scheme. Here, we study over 4000 reference strains and clinical isolates of *Shigella*, covering all serotypes, with both the current serotyping scheme and the standardised EnteroBase core-genome multilocus sequence typing scheme (cgMLST). The *Shigella* genomes are grouped into eight phylogenetically distinct clusters, within the *E. coli* species. The cgMLST hierarchical clustering (HC) analysis at different levels of resolution (HC2000 to HC400) recognises the natural population structure of *Shigella*. By contrast, the serotyping scheme is affected by horizontal gene transfer, leading to a conflation of genetically unrelated *Shigella* strains and a separation of genetically related strains. The use of this cgMLST scheme will facilitate the transition from traditional phenotypic typing to routine whole-genome sequencing for the laboratory surveillance of *Shigella* infections.

## Abstract (web summary)

Lab-based surveillance of *Shigella* has traditionally been based on serotyping but increasing availability of whole genome sequencing could enable higher resolution typing. Here, the authors apply a core genome multilocus sequence typing scheme to Shigella sequence data and describe its population structure.

## Introduction

*Shigella* belongs to the *Enterobacteriaceae* family, and causes bacillary dysentery, a common cause of diarrhoea in low- and middle-income countries. It has been estimated that this intracellular human pathogen, which is transmitted via the faecal-oral route with a very low infectious dose (10–100 cells), is responsible for over 210,000 deaths per year, mostly in children under the age of 5 years^1–3^. In high-income countries, *Shigella* infections also occur in travellers and in some high-risk groups, such as men who have sex with men (MSM) and Orthodox Jewish communities^2–5^. The morbidity of these infections is currently increasing due to growing resistance to antimicrobial drugs in these bacteria^2,3,5,6^. Since 2016, highly drug-resistant (i.e., resistant to at least ciprofloxacin, azithromycin and third-generation cephalosporins) *S. sonnei* isolates have been found in the US, England and Australia^3^.

Laboratory surveillance of *Shigella* infections was initiated several decades ago, and was facilitated by the adoption of a standardised *Shigella* typing scheme in the late 1940s^7^. This scheme, which is still in use today, is based on biochemical tests and serotyping (slide agglutination with typing sera directed against the different *Shigella* lipopolysaccharide O-antigens). It splits the *Shigella* genus into four serogroups (originally considered to be species): *Shigella dysenteriae*, *S. boydii*, *S. flexneri* and *S. sonnei*; these four serogroups are then subdivided into more than 50 serotypes. However, modern population genetics methods based on bacterial DNA sequences, such as multilocus sequence typing (MLST) analysis (which analyses the allelic profiles of — generally seven — housekeeping genes), and, more recently, core-genome single-nucleotide variant (cgSNV) analysis, have shown that shigellae form distinct lineages within the species *E. coli*, from which they emerged following the acquisition of a large virulence plasmid (VP) enabling the bacterium to invade intestinal cells^8–11^. In parallel, these host-restricted pathogens converged independently on the *Shigella* phenotype (non-motility, no decarboxylation of lysine, no use of citrate and malonate, and other characteristics, as reported by Pupo and coworkers^8^) through genome degradation.

More recent molecular approaches have shown that the current standardised *Shigella* typing scheme does not accurately reflect the population structure of this pathogen^8^. However, some molecular data have been taken into account in an update of the *Shigella* serotyping scheme. *S. boydii* serotype 13, for example, was withdrawn from the classification, because it was shown to belong to another species, *Escherichia albertii*, and lacked the VP^12,13^.

In an increasing number of countries, the laboratory surveillance of *Shigella* infections has now passed from conventional serotyping to real-time genomic surveillance^10,14^. The genomic methods used mostly target the O-antigen gene cluster (*rfb*) or the *S. flexneri* serotype-converting prophages, to ensure serotype specificity^14,15^. In addition, a set of 22 accessory genome genes was recently used to reassign *Shigella* serotypes to eight clusters^16^. Whilst these methods maintain backward compatibility between the genomic and serotyping data, they do not fully exploit the full resolution of genomic data. Hence, an extension of the MLST method to cover hundreds to thousands of core-genome genes has been developed. This high-resolution method, core-genome MLST (cgMLST), has been successfully used in the surveillance of many pathogens, including *Listeria monocytogenes*^17^ and *Salmonella enterica*^18^. Furthermore, cgMLST data are easy to interpret with clustering threshold methods, such as the hierarchical clustering (HierCC)^19^ implemented in EnteroBase^18,19^. However, until now, cgMLST has never been used for the comprehensive description of *Shigella* populations, and the utility of this method for the genomic surveillance of *Shigella* infections has not previously been assessed.

In this study, we analyse over 4000 genomes from phenotypically characterised *Shigella* strains representative of the global diversity of this pathogen. We aimed: (i) to resolve the population structure of *Shigella* using cgMLST, (ii) to create a dictionary of correspondence between cgMLST HC and serotyping data, (iii) to evaluate the performance of different in silico serotype prediction tools and (iv) to update the *Shigella* serotyping scheme by describing new serotypes. We demonstrate that the combination of cgMLST HC with *rfb* gene cluster analysis would enhance the laboratory surveillance of *Shigella* infections, while maintaining backward compatibility with the current serotyping scheme.

## Results

### Global population structure of S*higella*

We assembled and sequenced a collection of 317 *Shigella* strains chosen on the basis of their representativeness of the known diversity of *Shigella* populations (i.e., covering all serogroups and serotypes, and the different lineages or phylogroups of *S. sonnei* and *S. flexneri*). The genomic diversity of this “reference” dataset was further increased by adding another 81 publicly available *Shigella* genomes (these 81 genomes representing the “reference+” dataset). The 398 genomes studied were from strains belonging to the *S. flexneri* (*n* = 191), *S. dysenteriae* (*n* = 83), *S. boydii* (*n* = 80) and *S. sonnei* (*n* = 44) serogroups (Supplementary Table 4). We determined the wider phylogenetic context of these *Shigella* genomes by also analysing 95 *E. coli* genomes, including 27 EIEC from eight different EIEC genomic clusters and 68 (of the 72) strains from the ECOR collection, considered representative of the diversity of natural populations of *E. coli*^20^. We studied these 493 genomes by two different approaches: cgMLST and SNV-based clustering.

**Fig. 1:**
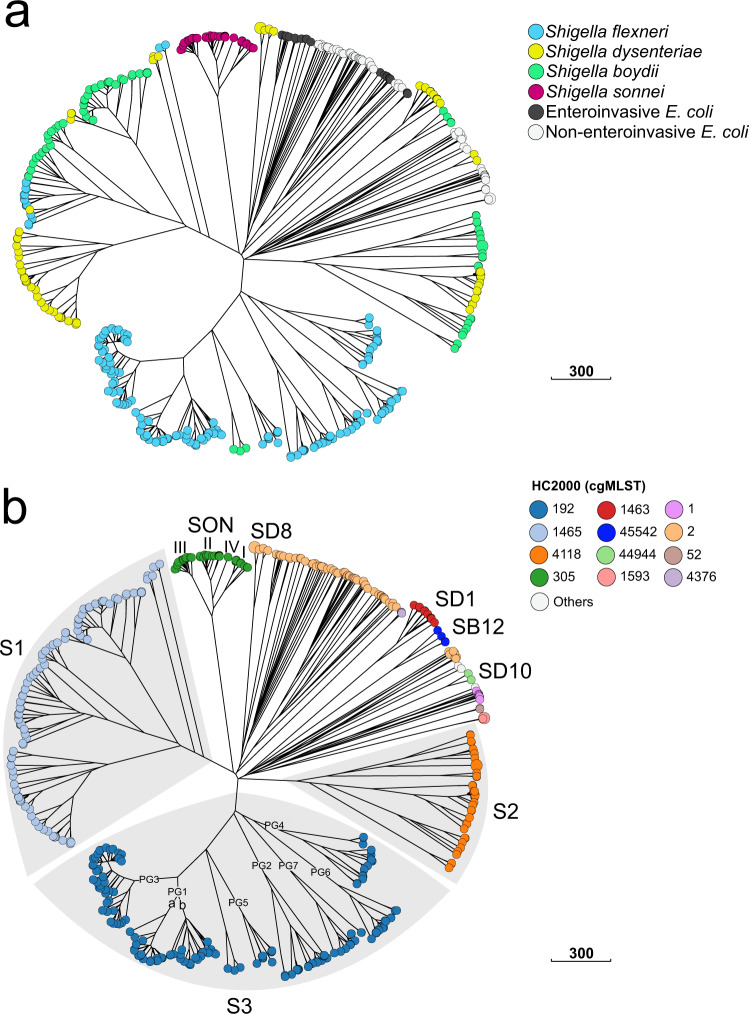
Population structure of *Shigella* spp. based on the cgMLST allelic differences between 493 *Shigella* and *E. coli* reference genomes. **a** A NINJA neighbour-joining (NJ) GrapeTree with tree nodes colour-coded by *Shigella* serogroup and *E. coli* pathovar. **b** A NINJA NJ GrapeTree with tree nodes colour-coded by HC2000 data. HC2000 clusters with fewer than two isolates are represented by white nodes. The different *Shigella* cgMLST clusters are labelled. For the SON cluster, the different genomic lineages of *S. sonnei* are indicated with Latin numerals. For the *S. flexneri* serotypes in cluster S3, the phylogenetic groups (PG1 to PG7) identified by Connor and coworkers^2^ are also indicated. The scale bar indicates the number of cgMLST allelic differences. The interactive version of the tree is publicly available from http://enterobase.warwick.ac.uk/ms_tree?tree_id=55118 and http://microreact.org/project/kP4HJjriDvAfTS4Ed3Avx8/01568b6f.

We used the EnteroBase *Escherichia*/*Shigella* cgMLST (2513 genes) scheme, which also assigns bacterial genomes to single-linkage hierarchical clusters (HCs) in real-time, at 13 fixed levels of resolution, ranging from HC0 (high-resolution clusters consisting of identical genomes with no allelic differences) to HC2350 (low-resolution clusters consisting of genomes with up to 2350 allelic differences). A previous evaluation by Zhou and coworkers^19^ found that in the genus *Escherichia*, cluster assignments at the HC2350, HC2000-HC1500, and HC1100 levels could be used to distinguish species, super-lineages and ST complexes, respectively. Since these levels of resolution are appropriate for routine surveillance, we predominantly used cgMLST HC2000 to HC400 levels to define the naturally, genetically discrete populations of *Shigella* (Fig. 1 and Supplementary Fig. 1).

**Fig. 2:**
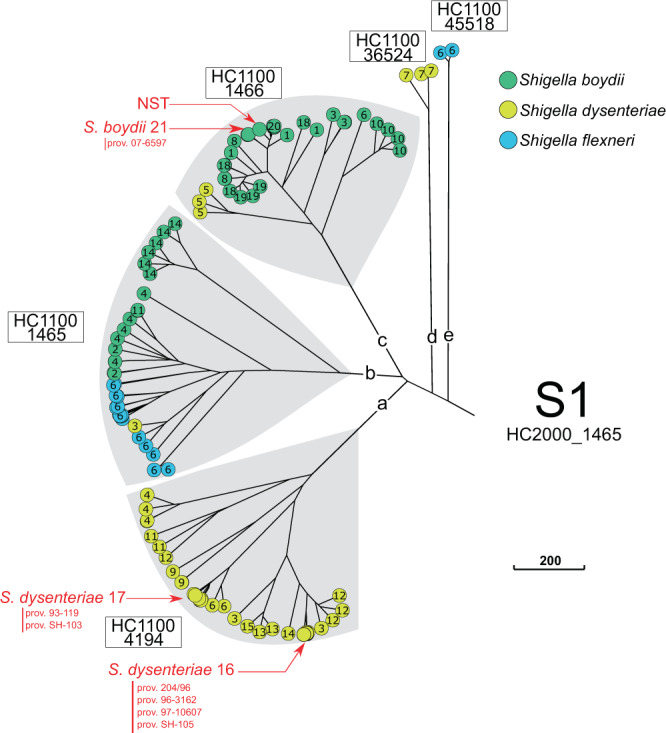
A NINJA NJ GrapeTree showing the population structure of the *Shigella* S1 cluster (HC2000_1465). This subtree is based on the tree shown in Fig. 1. The tree nodes are colour-coded by serogroup. The numbers within nodes indicate the serotype. HC1100 designation is indicated next to each subcluster. Novel and provisional (prov.) *Shigella* serotypes are also shown. NST non-serotypeable. The scale bar indicates the number of cgMLST allelic differences.

**Fig. 3:**
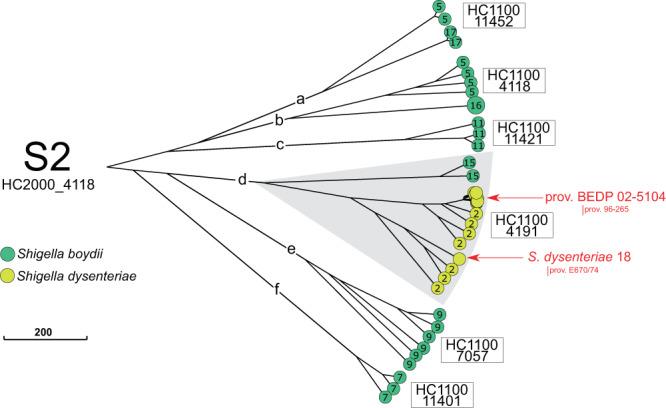
A NINJA NJ GrapeTree showing the population structure of the *Shigella* S2 cluster (HC2000_4118). This subtree is based on the tree shown in Fig. 1. The tree nodes are colour-coded by serogroup. The numbers within nodes indicate the serotype. HC1100 designation is indicated next to each subcluster. Provisional (prov.) *Shigella* serotypes are also shown. The scale bar indicates the number of cgMLST allelic differences.

**Fig. 4:**
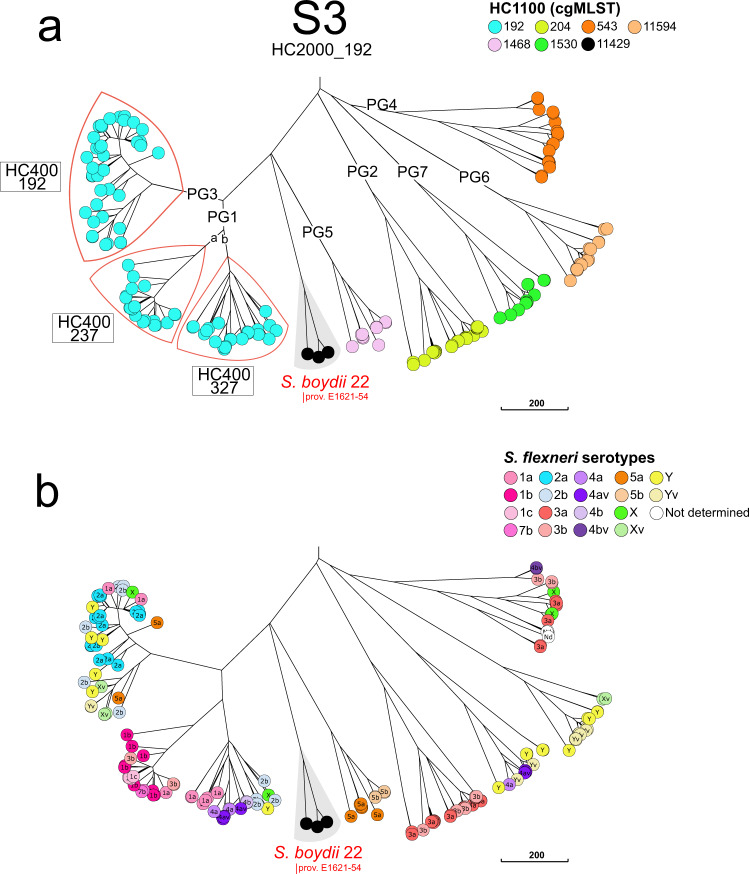
A NINJA NJ GrapeTree showing the population structure of the *Shigella* S3 cluster (HC2000_192). This subtree is based on the tree shown in Fig. 1. **a** The tree nodes are colour-coded by HC1100 data. The *S. flexneri* phylogenetic groups (PG) identified by Connor and coworkers^2^ are indicated. Some HC400 clusters are indicated to separate PG3 from PG1. *S. boydi* 22 (formerly prov. E1621-54) is shown. **b** The tree nodes are colour-coded by *S. flexneri* serotype. The scale bar indicates the number of cgMLST allelic differences.

Accordingly, by cgMLST, all 493 genomes belonged to the same HC2350 cluster (HC2350_1) defining the species *Escherichia coli* (Supplementary Data 1). As expected, all the *Shigella* and EIEC genomes contained the pathogenicity gene *ipaH*, whereas the ECOR genomes did not (Supplementary Fig. 2). A NINJA neighbour-joining (NJ) tree of core genomic allelic distances was generated with the dataset for the 493 *Shigella* and *E. coli* genomes (Fig. 1a). The differential contribution of the reference and reference+ datasets to *Shigella* population diversity is shown in Supplementary Fig. 2. Visual examination of the colour-coded HC2000 tree revealed that the *Shigella* genomes were grouped into eight different HC2000 clusters (Fig. 1b). Seven of these HC2000 clusters contained exclusively *Shigella* genomes. The eighth, HC2000_2, contained *S. dysenteriae* type 8 and *E. coli* (EIEC and ECOR) genomes. Four HC2000 clusters contained *Shigella* genomes from a single serotype: HC2000_305 (*S. sonnei*), HC2000_1463 (*S. dysenteriae* type 1), HC2000_44944 (*S. dysenteriae* 10), and HC2000_45542 (*S. boydii* 12). These clusters are referred to as SON, SD1, SD10 and SB12, respectively. Three clusters comprised multiple serogroups and serotypes: HC2000_1465, HC2000_4118, and HC2000_192, referred to as S1, S2 and S3, respectively (Fig. 2–4, Supplementary Notes sections “Genomic clustering of *Shigella* reference strains” and “Discrepancies with published studies”).

The HC2000 clusters could themselves be divided into five or more HC1100 subclusters (S1a-S1e, S2a-S2f and PG1-PG7). Of note, identical serotypes were found in different clusters and subclusters. For example, *S. boydii* 11 was found in S1b and S2c, *S. dysenteriae* 3 in S1a and S1b, and *S. flexneri* 6 in S1b and S1e (Table 1). Our analysis extends a polyphyletic nature to the majority of *S. flexneri* serotypes and subserotypes, found in S3 (Fig. 4). Each of these six HC1100 subclusters of S3 contained two or more *S. flexneri* serotypes, with identical serotypes found in different subclusters (Fig. 4b and Table 1). At a higher level of resolution, 10 *Shigella* serotypes were grouped within specific HC400 clusters, whereas the other serotypes remained mixed in HC400 clusters or split between two to seven HC400 clusters (Table 1). However, with HC1100 and HC400, we found, for S3, a correspondence between the clustering obtained with cgMLST and that obtained by Connor and coworkers^2^, who previously subdivided *S. flexneri* 1–5, X, Y into seven phylogenetic groups (PG1-PG7), based on a Bayesian analysis of population structure (Fig. 4a, Supplementary Notes section “Genomic clustering of *Shigella* reference strains”). Finally, we confirmed that biochemically atypical *S. boydii* 14 (aerogenic) and *S. dysenteriae* 3 (aerogenic and mannitol positive) strains^21,22^ – the status of which remained a matter of debate for decades – were genuine *Shigella* strains belonging to S1b (Supplementary Notes section “Aerogenic strains of *S. boydii* 14 and *S. dysenteriae* 3”). The atypical *S. dysenteriae* 3 strain was derived from a *S. flexneri* 6 strain following the acquisition of the *S. dysenteriae* 3 *rfb* gene cluster (see next section “Genomic analysis of the O-antigen gene cluster”).

**Table 1:** Distribution of the different *Shigella* serotypes in clusters S1, S2, and S3 of 398 *Shigella* reference and reference+ genomes according to their HC2000, HC1100, and HC400 data.

Cluster (HC2000)	Subcluster (HC1100)	HC400	Serotype^a^
S1 (1465)	S1a (4194)	4194	**SD3**, SD13, SD15
		14,114	SD11, SD12
		17,375	SD4
		35,330	SD3, SD12, SD14, SD16
		35,368	SD6, SD17
		44,956	SD9
		45,269	SD9
		45,271	SD11
	S1b (1465)	1465	SB2, SB4, **SB11**
		11,126	**SF6**
		11,341	**SF6**
		13,048	**SF6**
		17,342	**SF6**
		22,378	**SD3**, **SF6**
		41,808	SB14
		45,451	**SF6**
	S1c (1466)	1466	SB1, SB8, SB18, SB19, SB20, SB21
		45,284	SD5
		45,300	SB6, SB10
		45,420	SB3
	S1d (36524)	36,524	SD7
	S1e (45518)	45,518	**SF6**
S2 (4118)	S2a (11452)	11,452	SB17
		11,601	**SB5**
	S2b (4118)	4118	**SB5**
		11,449	SB16
	S2c (11421)	11,421	**SB11**
	S2d (4191)	4191	SD2, SD18
		11,444	SB15
		11,651	SD2, SD prov. BEDP 02-5104
		44,479	SD2
	S2e (7057)	11,413	SB9
		11,414	SB9
		30,095	SB9
		61,169	SB9
	S2f (11401)	11,401	SB7
S3 (192)	S3 (11429)	11,429	SB22
	PG1 (192)	237	**SF1a**, SF1b, SF1c, **SF3b**, SF7b
		327	**SF1a**, **SF2b**, **SF4a**, **SF4av**, SF4b, **SFX**, **SFY**
	PG2 (204)	204	**SF3a**
		544	**SF3a**, **SF3b**
		41,673	**SF3a**
	PG3 (192)	192	**SF1a**, SF2a, **SF2b**, **SF5a**, **SFX**, **SFXv**, **SFY**, **SFYv**
	PG4 (543)	543	**SF3a**, **SF3b, SFX**
		12,646	ND
		12,706	**SF3b**, SF4bv
		13,955	**SF3a**
		55,988	ND
	PG5 (1468)	1468	**SF5a**, SF5b
		11,593	**SF5a**
	PG6 (11594)	11,594	**SFY**, **SFYv**
		22,583	**SFY**
		41,322	**SFXv**
	PG7 (1530)	1530	**SFY**
		1538	**SF4a**, **SF4av**, **SFY**, **SFYv**
		13,203	**SFY**

^a^*SB* *S. boydii*, *SD* *S. dysenteriae*, *SF* *S. flexneri*, *ND* not determined; polyphyletic serotypes (i.e., present in different HC2000 or HC1100 clusters) are highlighted in bold.

**Fig. 5:**
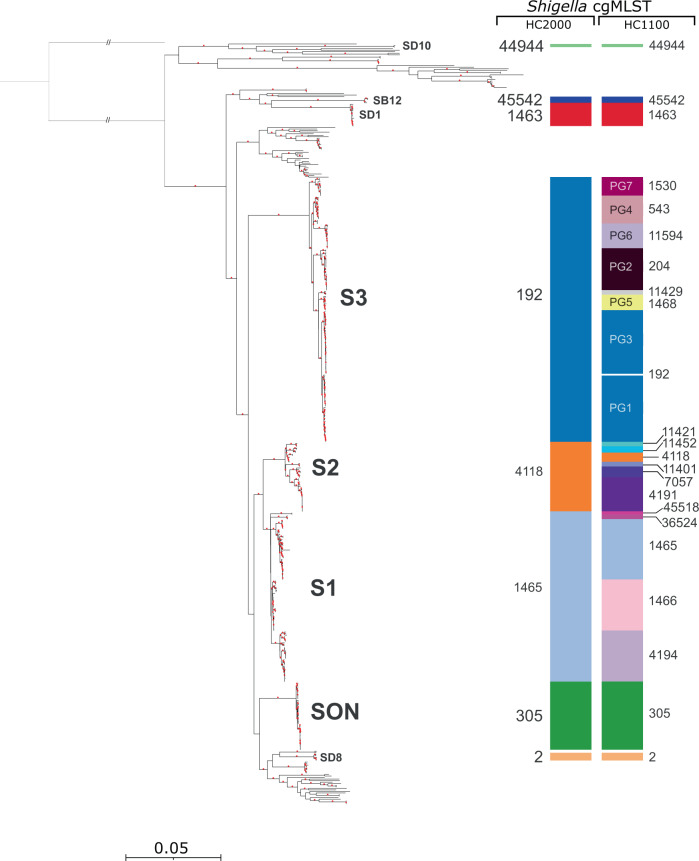
Population structure of 493 *Shigella* and *E. coli* reference genomes based on core-genome SNVs. This maximum-likelihood phylogenetic tree genomes are based on 92,688 core-genome single-nucleotide variants (SNVs). Nodes supported by bootstrap values ≥95% are indicated by red dots. Phylogenetic clades containing *Shigella* genomes are labelled with the same nomenclature (S1-S3, SON, SD1, SD8, SD10, and SB12) as in Fig. 1. All the *Shigella* genomes are also labelled on the right with cgMLST HC2000 and HC1100 data. The scale bar indicates the number of nucleotide substitutions per variable sites (SNVs).

We evaluated the accuracy of low-resolution cgMLST HCs for grouping *Shigella* genomes into different clusters of phylogenetic significance by employing another approach. We used the same dataset of 493 *Shigella* and *E. coli* genomes to infer two SNV-based ML trees: one based on 92,688 SNV sites (including recombinant sites) and the second on 5129 recombination-free SNVs. We compared these SNV-based clusterings (with strong bootstrap support) to clusters inferred by cgMLST HC. We found that the clustering of *Shigella* populations by HC2000 and by HC1100 were congruent with both core-genome SNV phylogenies (Fig. 5 and Supplementary Fig. 3). Even though cgMLST allelic distances (based on allelic changes counted as single genetic events, regardless of the number of SNVs involved) are reliable indicators of population structure, especially for routine surveillance tasks, SNVs are generally preferred for more detailed studies including long-term evolutionary dynamics. This likely accounts for the topological differences in the deep structures of cgSNV and cgMLST phylogenetic trees (Figs. 1 and 5 and Supplementary Fig. 3). Since the focus of this study was the use of cgMLST for routine surveillance of *Shigella* infections, resolution of these ancestral differences was not investigated further.

For confirmation of the robustness of the population structure of *Shigella* established by cgMLST analysis of our reference datasets, we also applied cgMLST to 3870 clinical *Shigella* isolates received by the FNRC-ESS between 2017 and 2020 (the “routine” dataset), in the framework of the French national surveillance programme for *Shigella* infections (see the methods section “Strain selection and typing”). All these isolates were characterised phenotypically, on the basis of biochemical reactions and serotyping. They belonged to *S. dysenteriae* (*n* = 53), *S. boydii* (*n* = 101), *S. flexneri* (*n* = 1555) and *S. sonnei* (*n* = 2161). All but one of these 3870 genomes were assigned to known serotype/HC2000/HC1100/HC400 combinations, without inconsistencies (Supplementary Data 1 and Supplementary Fig. 4). The exception was an HC1100_204 (PG2) *S. flexneri* isolate, grouped into a new HC400 cluster, HC400_11853. The stability of the cgMLST data was assessed by comparing allelic differences between pairs of *Shigella* isolates recovered from the same patient within months of each other (Supplementary Table 5). The routine isolates included 34 such pairs of isolates, each recovered from a single patient within 90 days (mean interval of 12 days between samplings). These pairs displayed between 0 and 5 (mean 1.5) cgMLST differences, for the 2513 alleles tested.

### Genomic analysis of the O-antigen gene cluster

The current typing scheme is mostly based on serotyping (targeting the variability of the O antigen, the polysaccharide part of lipopolysaccharide (LPS), the major bacterial surface antigen) and certain metabolic markers (Supplementary Notes “Genomic analysis of metabolic markers used in the current *Shigella* typing scheme”).

**Fig. 6:**
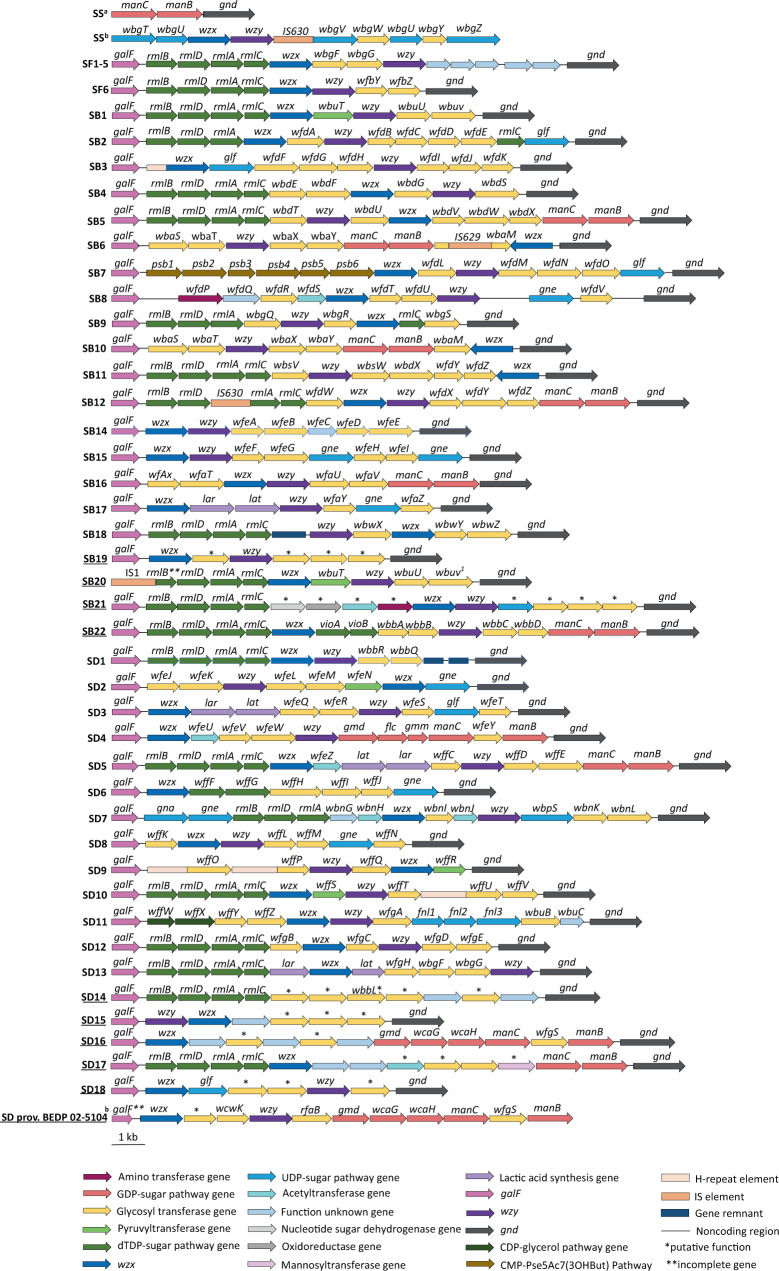
Representation of all previously and newly characterised *Shigella* O-antigen gene clusters. The names underlined correspond to those presented for the first time. Open arrows represent the location and orientation of putative genes. SS^a^ is a chromosomal remnant *rfb* from *S. sonnei*. A b in superscript indicates that the cluster is located on a plasmid. A 1 in superscript indicates that the gene may be truncated by an IS*1* in some strains.

**Fig. 7:**
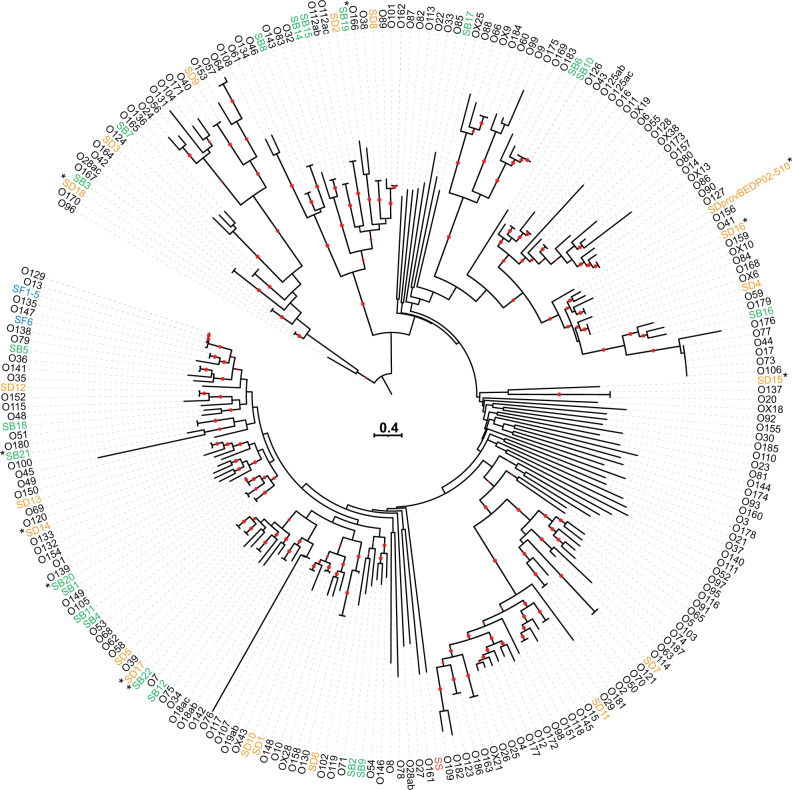
Phylogenetic tree for all *Shigella* and *E. coli* O-antigen gene clusters. The *Shigella* O-antigen gene clusters (*rfb*) characterised in this study are indicated by an asterisk. Nodes supported by bootstrap values ≥95% are indicated by red dots. *S. boydii rfb* genes are shown in green, S*. dysenteriae rfb* genes are shown in orange, *S. flexneri rfb* genes are shown in blue, and *S. sonnei rfb* genes are shown in red. The scale bar indicates the number of nucleotide substitutions per variable sites.

The *Shigella* O antigen gene cluster (*rfb*) is located on the chromosome, except in *S. sonnei*, in which it is located on a plasmid. The size of these *rfb* clusters ranges from 9 kb to 17 kb^23^. Consistent with their phylogenetic relationships, the *rfb* clusters of *Shigella* serotypes are identical, highly similar to, or adapted from *E. coli* O-antigen gene clusters, accounting for the many cross-agglutinations observed with the serotyping scheme^23^. We extended this analysis to populations not previously considered (Fig. 6) and showed that similarity between *E. coli* and *Shigella rfb* DNA sequences was also identified in *S. dysenteriae* 8 and *E. coli* O38, and in *S. boydii* 22 (formerly *S. boydii* provisional (prov.) E1621-54, see next section “Updating the *Shigella* typing scheme”) and *E. coli* O7 (Fig. 7). For both these *Shigella* serotypes, cross-agglutination with the corresponding *E. coli* serotypes was previously reported by Ewing^24^. Finally, the *rfb* from a new serotype, *S. dysenteriae* 16, originated from another species of *Escherichia*, *E. albertii* (serotype O2). Our genomic analysis also revealed very closely related *rfb* clusters present in the *Shigella* strains belonging to polyphyletic serotypes: identities of 13,192/13,622 (97%), gaps 3/13,622 (0%) between the *S. boydii* 11 S1b and S2c *rfb* gene clusters; and identities of 12,800/12,825 (99%) with no gap between the *S. dysenteriae* 3 S1a and S1b *rfb* gene clusters; identities of 10,431/10,448 (99%) with no gap between the *S. flexneri* 6 S1b and S1e *rfb* gene clusters.

### Updating the *Shigella* typing scheme

In recent decades, several provisional new serotypes of *S. dysenteriae* and *S. boydii* have been suggested^25,26^. However, the phylogenetic relationships between these different provisional serotypes have not been investigated. We characterised these relationships in detail (Supplementary Notes section “Updating the *Shigella* typing scheme”). We found that these provisional serotypes all belonged to the three main *Shigella* clusters, S1 to S3 (Fig. 2–4), and that many of those reported under different names were actually identical. After curation, the provisional serotypes found to belong to new serotypes were named *S. dysenteriae* 16–18, *S. boydii* 21 and *S. boydii* 22, and we propose to add them to the official serotyping scheme. All new *Shigella* serotypes possessed *rfb* clusters, similar to those found in known *E. coli* or *E. albertii* serotypes. All the reference strains for these new serotypes are now available from the *Collection de l’ Institut Pasteur* (CIP) or the National Collection of Type Cultures (NCTC). However, for *S. dysenteriae* prov. BEDP 02-5104, we propose to retain a provisional status, as our analysis (Supplementary Notes section “Updating the *Shigella* typing scheme”) suggests that this serotype is probably a *S. dysenteriae* 2 strain that has acquired an O-antigen-modifying plasmid from *E. coli* or *Citrobacter*, the expression of which has replaced the expression of the chromosomal genes. This plasmid also carries a raffinose operon, accounting for the use of this trisaccharide by *S. dysenteriae* prov. BEDP 02-5104, a very unusual trait in *S. dysenteriae*^26^.

### Performance of available in silico serotype prediction tools

**Fig. 8:**
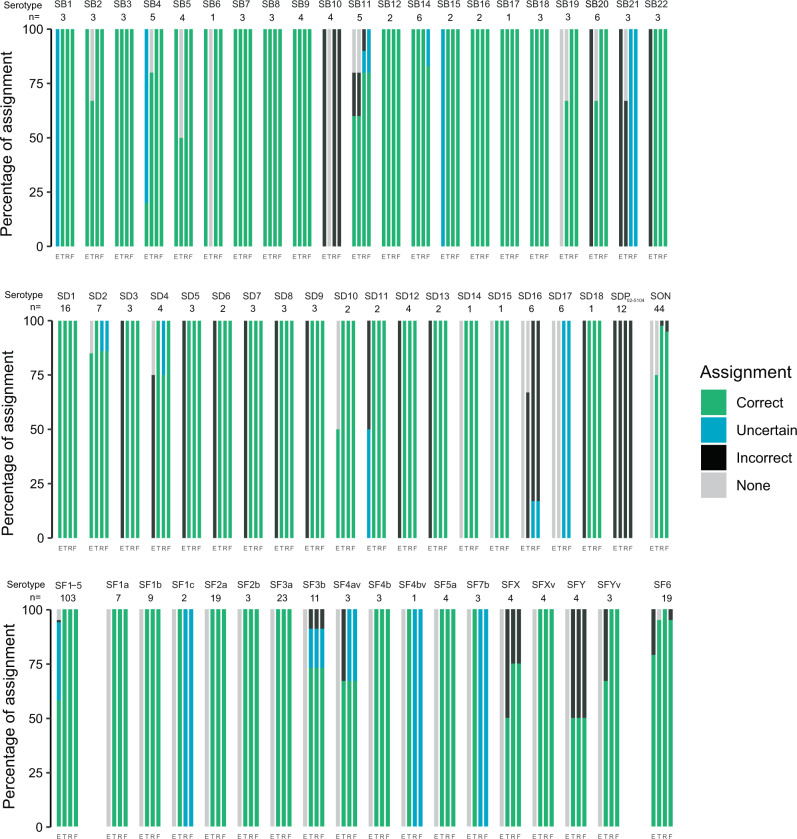
Graphical representation of the in silico serotyping results for 316 *Shigella* reference strains according to each serotype prediction tool. Stacked-bar chart displaying the results (percentage assignment) per tool for each *Shigella* serotype. E, the SeroPred tool implemented in EnteroBase^18^; T, ShigaTyper^14^; R, ShigEiFinder^16^ using short reads; F, ShigEiFinder using SPAdes assemblies. SB *S. boydii*, SD *S. dysenteriae*, SON *S. sonnei*, SF *S. flexneri*. A correct assignment means that the predicted serotype was the same as the phenotypically determined serotype. An uncertain assignment means that the correct serotype was listed among others or, for ShigEiFinder that only the cluster level (Cs1 to Cs3) was obtained. An incorrect assignment means that the predicted serotype was different from the phenotypically determined serotype. “None” means that no serotype was predicted. More detailed results can be found in Supplementary Table 6.

In silico serotyping tools have been developed by various groups, and can be used to maintain links with the current *Shigella* serotyping system. We assessed the performances of the three tools currently available: the “SeroPred” tool^18^ (implemented in EnteroBase), ShigaTyper^14^ (using a short-read mapping approach), and ShigEiFinder^16^ (using either short reads or assemblies) with our 316 reference strain genomes with known serotype designations (Supplementary Tables 4, 6 and 7). When all *Shigella* serotypes and subserotypes were considered (SeroPred does not discriminate the serotypes and subserotypes of *S. flexneri* 1–5, X and Y), the rates of correct assignment were 80.1% (253/316), 84.5% (267/316) and 83.9% (265/316) for ShigaTyper, ShigEiFinder (short reads) and ShigEiFinder (assemblies), respectively. ShigEiFinder gave the best serotype prediction results, whether based on short reads or assemblies, particularly for *S. sonnei* and *S. boydii* (Fig. 8 and Supplementary Table 6). However, 100% of the strains belonging to *S. boydii* 10 and to the new serotypes *S. dysenteriae* 17 and *S. boydii* 21, and 14–20% of the strains from *S. boydii* 11, *S. boydii* 14 and *S. dysenteriae* 2 were incorrectly assigned by ShigEiFinder. Furthermore, all the genomes belonging to *S. dysenteriae* prov. BEDP 02-5104 were incorrectly predicted to be *S. dysenteriae* 2, while 83% of the strains from the new serotype *S. dysenteriae* 16 were incorrectly predicted to be *S. dysenteriae* prov. 96–265, and 17% were not assigned.

**Fig. 9:**
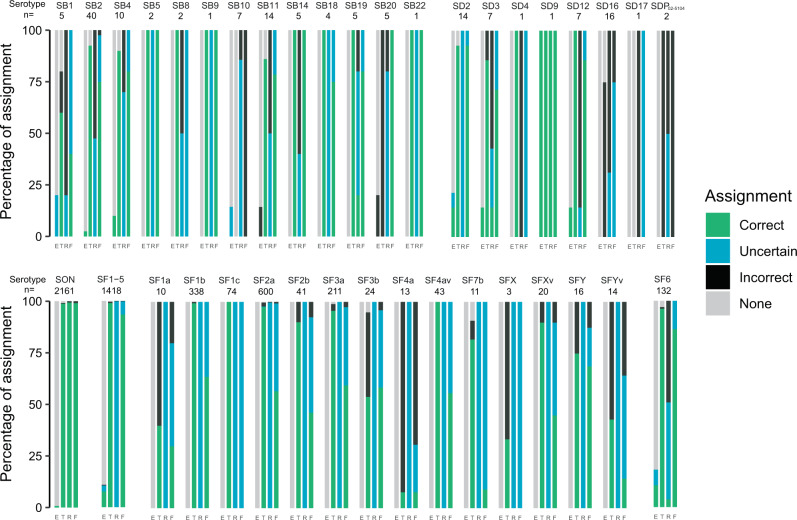
Graphical representation of the in silico serotyping results for 3861 *Shigella* “routine” isolates according to each serotype prediction tool. Stacked-bar chart displaying the results (percentage assignment) per tool for each *Shigella* serotype. E, the SeroPred tool implemented in EnteroBase^18^; T, ShigaTyper^14^; R, ShigEiFinder^16^ using short reads; F, ShigEiFinder using SPAdes assemblies. SB *S. boydii*, SD *S. dysenteriae*, SON *S. sonnei*, SF *S. flexneri*. A correct assignment means that the predicted serotype was the same as the phenotypically determined serotype. An uncertain assignment means that the correct serotype was listed among others or for ShigEiFinder that only the cluster level (Cs1 to Cs3) was obtained. An incorrect assignment means that the predicted serotype was different from the phenotypically determined serotype. “None” means that no serotype was predicted. More detailed results can be found in Supplementary Table 7.

When analysing the 3861 “routine” genomes from isolates with known serotype designations (9/3870 could not be serotyped, Supplementary Table 4), 96.2% (3713/3861), 55.9% (2157/3861) and 80.8% (3120/3861) of the strains were correctly assigned by ShigaTyper, ShigEiFinder (short reads) and ShigEiFinder (assemblies), respectively. ShigEiFinder performed less well than ShigaTyper with this routine dataset (Fig. 9 and Supplementary Table 7). In particular, most of the results obtained with short reads were “uncertain” (i.e., assignment to a *Shigella* cluster, but not to a serotype). ShigEiFinder uses 22 accessory genome (non-O antigen) genes to assign *Shigella* genomes to eight clusters, with the serotype assignment based on the *Shigella* serotype-specific *wzx*/*wzy* genes (and O-antigen modification genes for *S. flexneri*). The inferior performance of ShigEiFinder can be explained by the low read coverage of the AT-rich *rfb* gene cluster for the “routine” genomes. Our public health sequencing platform (P2M) uses the Nextera XT kit (Illumina, San Diego, CA, USA), according to an in-house protocol, for library preparation, and transposase-based library generation is known to be biased against AT-rich sequences^14^.

## Discussion

We present here a broad overview of the population of *Shigella*. The hierarchical clustering of cgMLST data and a cgSNV analysis showed that *Shigella* strains belong to eight phylogenetically distinct clusters within the *E. coli* species. Our results are consistent with previous studies suggesting multiple origins of the “*Shigella*” phenotype^8,27^. However, the higher resolution of cgMLST and comprehensive sampling from thousands of phenotypically characterised isolates and reference strains covering all serotypes – including provisional serotypes and atypical strains – made it possible to complete, and in some cases amend, the *Shigella* population structure obtained in previous studies.

The 70-year-old *Shigella* typing scheme, which is still in use today, was based on biochemical characteristics, antigenic relationships, and tradition^7^. We show here that, unlike cgMLST, this scheme does not reflect the natural population structure of these bacteria. In particular, the *Shigella* serogroups/species are artificial constructs developed from data for antigen and metabolic markers affected by IS element inactivation or horizontal gene transfer. The presence and expansion of large numbers of ISs in *Shigella* genomes can disrupt coding sequences or cause genome rearrangements and deletions, thereby altering the nature of both the O-antigen and the rare phenotypic markers identified in this bacterium with weak metabolic activity^9^. For example, *S. boydii* 6 and 20 arose in subcluster 1c following the insertion of a single IS within the *rfb* cluster of *S. boydii* 10 and 1, respectively. Serotype diversification, which is observed mostly in clusters S1 to S3, also occurs via horizontal gene transfer of the O-antigen-encoding *rfb* cluster from *Escherichia* or *Shigella* donors^8,28^. Horizontal gene transfer outside of the *rfb* cluster can also alter the serotype of a strain, as illustrated particularly clearly by the S3 cluster. All the *S. flexneri* strains in this cluster share the same O-antigen backbone structure, and their serotypes are determined by glucosylation and/or O-acetylation modifications to the O-antigen tetrasaccharide repeat, conferred by prophage-encoded *gtr* and/or *oac* genes, respectively^15^. Plasmid-mediated serotype conversion by the O-antigen phosphoethanolamine transferase gene (*opt*) has also been reported in *S. flexneri*^15^. Each of the seven *S. flexneri* phylogenetic groups (PGs) described by Connor and coworkers^2^, based on a cgSNV analysis, contained two or more of these serotypes. This serotyping method does not reflect the genetic relatedness between *Shigella* isolates, and has a number of other disadvantages, including being time-consuming, having intra- and interspecies cross-reactivity, not allowing for typing of rough strains and new serotypes^14,25^. Therefore, modern laboratory surveillance of *Shigella* infections should now be based on phylogenetically relevant methods rather than simply on molecular or in silico serotyping^10,14–16^.

The cgMLST HC analysis provides, in a single step, a wide range of fixed clustering levels, from HC0 (no allelic differences allowed) to HC2350 (maximum of 2350 allelic differences allowed), with a standard and stable nomenclature. In our study, cgMLST HC analysis at low levels of resolution (HC2000 to HC400) provided sufficient resolution to monitor the trends in globally circulating *Shigella* types. The different clusters of S*higella* can be identified with HC2000. Using HC1100 and, in certain instances HC400, can provide added detail needed to reveal additional subclusters. This is particularly interesting for S3, which contains the *S. flexneri* 1–5, X, and Y serotypes generated via horizontal gene transfer rather than by vertical descent. We therefore recommend integrating the seven phylogenetic groups (PG1-PG7) described for *S. flexneri* into routine genomic surveillance for *S. flexneri*. These PGs can be easily inferred from cgMLST HC1000/HC400; it is even possible to obtain up to eight groups (after subdividing PG1 into PG1a and PG1b).

High-resolution subtyping (at strain level) is needed for the most frequent — and often genetically homogeneous — *Shigella* serotypes, such as *S. sonnei* and *S. flexneri* 2a, for the identification of a single-source outbreak or to follow up an epidemic strain. Such studies have traditionally employed cgSNV analysis^4,6^. The high-resolution levels of EnteroBase cgMLST, HC5 and HC10, have been used to detect foodborne outbreaks caused by *Salmonella*^18,29,30^ and enterohaemorragic *E. coli*^31^. Our study aimed to assess the EnteroBase cgMLST tool as a replacement for the current serotyping scheme. However, this study did not set out to assess the performance of HC5 and HC10 for the high-resolution subtyping of prevalent *Shigella* populations. For these purposes, a robust and practical hierarchical SNV-based genotyping scheme developed for *S. sonnei* by Hawkey and coworkers^3^ provided more resolution than even the high-resolution levels of EnteroBase cgMLST. For example, unlike the hierarchical SNV-based genotyping scheme, HC5 (and even HC10) could not easily identify the internationally recognised *S. sonnei* epidemic strains, such as those resistant or highly resistant to multiple drugs that have recently spread among MSM globally. These epidemic strains were split into multiple HC5 and HC10 clusters with non-informative numbers, in contrast to the findings with the SNV-based genotyping scheme (which indicates the lineage, clade, subclade, and, when necessary, higher-resolution genotypes and strains of epidemiological interest, with a human-readable alias; e.g., 3.6.1.1.2 (CipR.MSM5)). Despite the utility of our proposed EnteroBase cgMLST-based approach in routine surveillance, it has clear limitations for the global surveillance of *S. sonnei* infections.

Despite the aforementioned limitations in select circumstances, the use of such standardised cgMLST HC data makes it possible to query EnteroBase, which contains over 160,000 *E. coli*/S*higella* genomes, to identify strains with similar HC types at any level of resolution. This can facilitate the investigation of unusual types of *Shigella* (e.g., our analysis of the provisional serotypes) or outbreaks with an international dimension^32^.

Importantly, the use of cgMLST HC data in surveillance should be paired with in silico serotyping to achieve backward compatibility with the current serotyping scheme. This is crucial for the maintenance of international surveillance with laboratories that cannot currently afford genomic surveillance and to prevent disjunction with the seven decades of serotyping data accumulated worldwide. To this end, we found that ShigaTyper^14^ and ShigEiFinder^16^ were promising tools. However, both require optimisation in the choice of molecular targets for certain serotypes. The complete set of *rfb* sequences provided by our study would be helpful for improving them. ShigEiFinder also requires optimisation of the read mapping thresholds used to determine the presence or absence of the O-antigen genes.

In conclusion, by studying >4000 serotyped reference strains and routine isolates covering the overall diversity of *Shigella*, we were able to demonstrate that cgMLST is a robust and portable genomic method revealing natural populations for this pathovar of *E. coli*. The cgMLST method has substantial added value in the framework of the laboratory monitoring of *Shigella*, as it prevents genetically unrelated strains from being conflated, and genetically related strains from being separated. However, we strongly recommend combining cgMLST with in silico serotyping to maintain backward compatibility with the current *Shigella* serotyping scheme.

## Methods

### Strain selection and typing

In total, 4187 *Shigella* reference strains and clinical isolates were studied (Supplementary Data 1). Two datasets were used. The first dataset – the “reference” dataset – consisted of 317 *Shigella* reference strains covering all the known serotypes – including provisional serotypes – from various geographic locations (53 countries on four continents) and time periods (1913 to 2019). These strains originated from the French National Reference Centre for *E. coli*, *Shigella*, and *Salmonella* (FNRC-ESS), Institut Pasteur, Paris, except for 11 strains belonging to provisional serotypes and provided by the Public Health Agency of Canada, Winnipeg, Canada; the Centers for Disease Control and Prevention, Atlanta, USA; the Tokyo Metropolitan Research Laboratory of Public Health, Tokyo, Japan; and the International Centre for Diarrhoeal Disease Research, Bangladesh, Dhaka. This “reference” dataset included 44 *S. sonnei* from four different lineages^33^, 16 *S. dysenteriae* type 1 (ref. ^34^) and 98 *S. flexneri* serotypes 1–5, X and Y belonging to the seven phylogenetic groups (PGs) described previously^2,5^.

The second dataset – the “routine” dataset – consisted of 3870 clinical isolates received and sequenced by the FNRC-ESS between 2017 and 2020 in the framework of the French national surveillance programme for *Shigella* infections. This programme is based on a voluntary laboratory-based network consisting of ~1000 clinical laboratories located in mainland France and its overseas territories in South America, the Caribbean and Indian Ocean, which send ~1000–1200 *Shigella* isolates to the FNRC-ESS each year (only 600 in 2020, due to the COVID pandemic). This surveillance system has been estimated to detect 50–60% of laboratory-confirmed *Shigella* infections in France^35^. Between 2017 and 2020, 3942 clinical isolates were received and sequenced at the FNRC-ESS, and we included the 3870 genomes that passed the quality control criteria of EnteroBase. All these strains and isolates were thoroughly characterised with a panel of biochemical tests and serotyped by slide agglutination assays according to standard protocols, as previously described^36^. Additional typing sera against KIVI 162 and SH-105 were provided by the International Centre for Diarrhoeal Disease Research, and the Public Health Agency of Canada, respectively.

### DNA extraction and sequencing

Total DNA was extracted with the Wizard Genomic DNA Kit (Promega, Madison, WI, USA), the Maxwell 16-cell DNA purification kit (Promega) or the MagNA Pure DNA isolation kit (Roche Molecular Systems, Indianapolis, IN, USA), in accordance with the manufacturer’s recommendations. The 4187 strains and isolates were sequenced with different Illumina platforms. FqCleanER version 3.0 (https://gitlab.pasteur.fr/GIPhy/fqCleanER) was used to eliminate adaptor sequences^37^, correct sequencing errors^38^ and discard low-quality reads. Assemblies were generated with SPAdes^39^ version 3.15.

### Other studied genomes

With the aim of capturing the broadest possible diversity of S*higella* populations, we searched the *E. coli*/*Shigella* database in EnteroBase^18^, and selected 81 additional *Shigella* genomes (“reference+” dataset) not originating from the Institut Pasteur (Supplementary Methods section “Other studied genomes”). We included 27 enteroinvasive *E. coli* (EIEC) and 68 *E. coli* strains from the *E. coli* reference (ECOR) collection (Supplementary Methods section “Other studied genomes”), to place our *Shigella* genomes in the phylogenetic context of the broader diversity of *E. coli*. We also used the closed PacBio sequences available for all *Shigella* serotypes and described by Kim and coworkers^40^, to study the genetic organisation of the *rfb* gene cluster or various operons described in the “Gene analyses” section. However, these closed genomes were not included in the cgMLST analysis, as they were not edited with short reads and the numerous indels in the homopolymers therefore altered the allelic distances (Supplementary Table 1).

### Characterisation of the O-antigen gene clusters

The *Shigella* O-antigen biosynthetic gene (*rfb*) cluster was analysed after extraction of the region between the housekeeping genes *galF* (encoding UTP-glucose-1-phosphate uridylyltransferase) and *gnd* (encoding 6-phosphogluconate dehydrogenase), which are known to flank the *rfb* cluster^28^. Newly identified *rfb* clusters were annotated on the basis of a previously annotated closely matched *E. coli* cluster in the NCBI BLASTn nucleotide collection (nr/nt) database (100% coverage and at least 99% identity) or with ORFfinder (https://www.ncbi.nlm.nih.gov/orffinder/) when no matching cluster was found in the NCBI BLAST database (https://blast.ncbi.nlm.nih.gov/Blast.cgi). The GenBank accession codes of all the *Shigella rfb* clusters are listed in Supplementary Table 2. We also used three tools for in silico serotyping: SeroPred, the serotype prediction tool implemented in EnteroBase^18^, ShigaTyper^14^ (using short reads), and ShigEiFinder^16^ (using both short reads and assemblies).

### Phylogenetic analyses

We used the *Escherichia*/*Shigella* cgMLST scheme (“cgMLST V1”) implemented in EnteroBase^18^ to study our genomic datasets. This scheme is based on 2513 single-copy orthologous genes found in the soft-core genome of a representative set of *Escherichia* and *Shigella* genomes^18^. The cgMLST sequence types (cgSTs) consist of a combination of up to 2513 integers, each representing a sequence variant (allele) of a gene, accounting for missing data (gene missing, allele not called due to short-read misassemblies). Genetic distances between genomes were calculated from the number of shared cgMLST alleles. The cgMLST trees were inferred with the NINJA neighbour-joining algorithm, present in the “cgMLST V1+HierCC V1” scheme of EnteroBase. We visualised the cgMLST data with GrapeTree^41^.

These bacterial genomes were also assigned to clusters at multiple levels of resolution, by a single-linkage hierarchical clustering pipeline (pHierCC), implemented in the “cgMLST V1+HierCC V1” scheme, and described in detail by Zhou and coworkers^19^. Each genome was assigned to 13 clusters – one for each level of resolution (HC0, HC2, HC5, HC10, HC20, HC50, HC100, HC200, HC400, HC1100, HC1500, HC2000 and HC2350) – with stable numbers. In EnteroBase, genomes equidistant from multiple clusters were assigned to the oldest cluster (i.e., with the smallest HC number) in order to ensure the long-term stability of the nomenclature. Genomes with >3% of missing data were also processed separately by pHierCC to minimise the impact of missing data on clustering^19^.

We also performed cgSNV analysis to assess the phylogenetic relationships of 398 *Shigella* (317 from the “reference” dataset and 81 from the “reference+” dataset) and 95 *E. coli* (68 ECOR and 27 EIEC) strains. An *Escherichia fergusonii* genome (RHB19-C05, GenBank accession no. GCF_013892435.1) was used as an outgroup for the cgSNV analysis. The paired-end reads and simulated paired-end reads were mapped onto the reference genome of *E. coli* K12-MG1655 (GenBank accession no. NC_000913.3) with Snippy version 4.6 (https://github.com/tseemann/snippy). We used Snippy default settings, except–mincov 4–minfrac 0.75, to ensure consistency with previous work^34^. Gubbins^42^ version 2.4.1 (default settings, except -f 30) was used to identify regions of recombination within the core genome alignment. Two alignments were used for phylogenetic inference, one of 92,688 SNVs (including recombinant sites) and a second alignment of 5129 SNVs (recombination removed). We generated maximum-likelihood (ML) phylogenetic trees with RAxML-NG^43^ version 1.0.1 using the general time-reversible (GTR) model of nucleotide substitution with a gamma model of between-site heterogeneity rate (GTR+G) and 100 bootstrap iterations. The best-scoring ML tree of the 20 replicates was midpoint-rooted and visualised with interactive tree of life (iTOL)^44^ version 6 (https://itol.embl.de).

A phylogenetic tree of *rfb* sequences was constructed with the sequences from 43 *Shigella* (Supplementary Table 2) and 196 *E. coli* strains from DebRoy and coworkers^28^. The *Shigella rfb* sequences were trimmed to ensure the same start and end points as for the *E. coli rfb* sequences from DebRoy and coworkers^28^. A sequence alignment was generated with MEGA X^45^ version 10.2.1, using ClustalW with default settings. A ML phylogeny was created with RAxML-NG^43^ version 1.0.1, using the GTR+G model and 100 bootstrap replicates. The ML tree with the best score of the 20 replicates was midpoint-rooted and visualised with iTOL^44^ version 6 (https://itol.embl.de).

### Gene analyses

The presence of the *ipaH* gene, a multicopy gene unique to *Shigella* and EIEC^46^, the presence and structure of the mannitol (*mtl*)^47^, raffinose (*raf*)^48^, and tryptophanase (*tna*) operons^49^ and the type of the O-antigen gene cluster (*rfb*) were determined using SPAdes assemblies with NCBI BLASTn version 2.10.1 (https://blast.ncbi.nlm.nih.gov/Blast.cgi). The target sequences are described in Supplementary Table 3.

### Data collection

The data were entered into an Excel (Microsoft) version 15.41 spreadsheet (Supplementary Data 1).

### Reporting summary

Further information on research design is available in the Nature Research Reporting Summary linked to this article.

## Supplementary information

Supplementary Information

Description of Additional Supplementary Files

Supplementary Data 1

Reporting Summary

## Data Availability

Short-read sequence data were submitted to EnteroBase (https://enterobase.warwick.ac.uk/) and to the European Nucleotide Archive (ENA, https://www.ebi.ac.uk/ena/) under study numbers PRJEB44801, PRJEB2846, and PRJEB2128. Other whole-genome sequences analysed during the study are available from ENA (https://www.ebi.ac.uk/ena/), NCBI RefSeq (https://www.ncbi.nlm.nih.gov/refseq/), DDBJ (https://www.ddbj.nig.ac.jp/index-e.html), and GenBank (https://www.ncbi.nlm.nih.gov/genbank/). All the accession numbers of the genomes used in this study are listed in Supplementary Data 1. The GrapeTree of 493 *Shigella* and *E. coli* reference genomes is publicly available from EnteroBase (http://enterobase.warwick.ac.uk/ms_tree?tree_id=55118) and from Microreact (https://microreact.org/project/kP4HJjriDvAfTS4Ed3Avx8/01568b6f). The nucleotide sequences of the *Shigella rfb* clusters were submitted to GenBank (https://www.ncbi.nlm.nih.gov/genbank/) under accession numbers MZ286364-MZ286394, MZ303046, MF322747-MF322752, and MF322754. The accession numbers for the individual *rfb* sequences are given in Supplementary Table 2.

## Code availability

All custom scripts can be found at https://github.com/imanyass/Shigella_population_2021.
