# Supplementary material for: Population structure analysis and laboratory monitoring of Shigella by core-genome multilocus sequence typing
Source: Nat Commun. 2022 Jan 27;13:551. doi: 10.1038/s41467-022-28121-1 (PMC8795385; doi:10.1038/s41467-022-28121-1)
Supplement: Supplementary file 1 — Supplementary Information [file 41467_2022_28121_MOESM1_ESM.pdf]

Supplementary Information

Population structure analysis and laboratory monitoring of *Shigella* by core-genome multilocus sequence typing

Iman Yassine, Sophie Lefèvre, Elisabeth E. Hansen, Corinne Ruckly, Isabelle Carle, Monique Lejay-Collin, Laëtitia Fabre, Rayane Rafei, Dominique Clermont, Maria Pardos de la Gandara, Fouad Dabboussi, Nicholas R. Thomson, François-Xavier Weill.

Supplementary Methods

Other studied genomes

EnteroBase[1] was queried on 11 November 2020, to identify genomes with new HC1100/HC400 combinations (i.e., not present in our reference and routine datasets) for a given serotype, determined by *in silico* analysis of the *rfb* cluster. We selected at least one genome for each unique combination and a maximum of five if the genomes concerned appeared to come from epidemiologically unrelated isolates. In addition, if a serotype was represented by only one strain in our collection, we selected another genome from EnteroBase, when possible. This selection resulted in 81 *Shigella* genomes (reference+ dataset). The 27 enteroinvasive *E. coli* (EIEC) included in this study belonged to the various previously described EIEC clusters[2–5]. We discarded four (ECOR 7, 23, 32, 43) of the 72 *E. coli* strains from the ECOR collection[6] from this study due to discrepant results for MLST and/or Clermont typing between the studies of Galardini and coworkers[7], Clermont and coworkers[8], and EnteroBase[1]. These 81 additional *Shigella* genomes, the 68 *E. coli* genomes from the ECOR collection, and the 27 EIEC genomes are listed in Supplementary Data 1.

Supplementary Notes

Genomic clustering of *Shigella* reference strains

One *Shigella* reference strain originally present in our collection (UE 95-1589)[9] and previously described as *S. boydii* 7 was actually *Escherichia albertii* (HC2350_1596). It did not contain the invasion plasmid antigen gene *ipaH,* instead bearing the intimin *eae* gene, a pathogenicity gene present in enteropathogenic *E. coli* (EPEC) and enterohaemorrhagic *E. coli* (EHEC). This strain, and the *S. boydii* 13 strains, now reclassified as *E. albertii*[10] (HC2350_1596), were not, therefore, included in this study.

Shigella genomes were grouped into eight different HC2000 clusters (Fig. 1B). Four HC2000 clusters contained *Shigella* genomes from a single serotype: HC2000_305 (SON) with *S. sonnei*, HC2000_1463 (SD1) with *S. dysenteriae* type 1, HC2000_44944 (SD10) with *S. dysenteriae* 10, and HC2000_45542 (SB12) with *S. boydii* 12, and three clusters — HC2000_1465 (S1), HC2000_4118 (S2), and HC2000_192 (S3) — consisted of multiple serogroups and serotypes.

The first of these clusters, HC2000_1465, contained various serotypes of *S. dysenteriae* (3, 4-7, 9, 11-15, provisional (prov.) 93-119, prov. SH-103, prov. 97-10607, prov. SH-105, prov. 96-3162 and prov. 204/96); *S. boydii* (1-4, 6, 8, 10, 11, 14, 18-20, and prov. 07-6597); and *S. flexneri* type 6 (Fig. 2). This was consistent with Cluster 1 described by Pupo and coworkers[11] in their MLST analysis of 46 diverse *Shigella* strains. The HC2000_1465 cluster, named S1, can be divided into five HC1100 clusters (Fig. 2). Only the HC1100_36524 cluster (subcluster S1d) contained strains from a single serotype, *S. dysenteriae* 7. The HC1100_45518 cluster (S1e) contained only *S. flexneri* 6 strains, but most strains from this serotype were in another

HC1100 cluster, HC1100_1465 (S1b), along with *S. dysenteriae* 3 (Supplementary Notes section "Aerogenic strains of *S. boydii* 14 and *S. dysenteriae* 3") and various serotypes of *S. boydii*. The HC1100_1466 cluster (S1c) contained *S. dysenteriae* 5 and various serotypes of *S. boydii*. Finally, the HC1100_4194 cluster (S1a) included only *S. dysenteriae* strains, but from diverse serotypes. *S. dysenteriae* 3 was found in two different S1 subclusters, S1a and S1b. At a higher level of resolution, four *Shigella* serotypes were grouped within specific HC400 clusters, whereas the other serotypes were split between two to seven HC400 clusters (Table 1).

The second cluster, HC2000_4118, comprised various serotypes of *S. dysenteriae* (2, prov. E670/74, prov. 96-265, and prov. BEDP 02-5104) and *S. boydii* (5, 7, 9, 11, 15-17) (Fig. 3). This cluster, consisting exclusively of indole-positive strains (Supplementary Notes sections "Genomic analysis of metabolic markers used in the current *Shigella* typing scheme"), corresponds to the Cluster 2 described by Pupo and coworkers[11]. The HC2000_4118 cluster, hereafter referred to as S2, could be divided into six distinct HC1100 clusters (Fig. 3). Five of these HC1100 clusters contained exclusively *S. boydii*; the sixth, HC1100_4191 (subcluster S2d), contained *S. boydii* 15 and all the *S. dysenteriae* serotypes found in S2. Three HC1100 clusters contained a single serotype: HC1100_11401 (S2f) for *S. boydii* 7, HC1100_7057 (S2e) for *S. boydii* 9, and HC1100_11421 (S2c) for *S. boydii* 11. This last serotype was also found in the S1 cluster (S1b subcluster). At higher resolution, it was possible to assign some serotypes to a particular HC400 cluster. This was the case for *S. boydii* 16 (HC400_11449) and *S. boydii* 17 (HC400_11452). However, at this level of resolution, other serotypes were split between two to four clusters (Table 1).

The third cluster, HC2000_192, comprised *S. boydii* prov. E1621-54 (now proposed as *S. boydii* 22, Supplementary Notes section "Updating the *Shigella* typing scheme) and all serotypes and subserotypes of *S. flexneri*, with the exception of *S. flexneri* 6, which grouped in S1 (Fig. 4). This cluster seems to correspond to the Cluster 3 reported by Pupo and coworkers[11], except that *S. boydii* 12 rather than *S. boydii* prov. E1621-54 was reported in Cluster 3 in this previous study (Supplementary Notes section "Discrepancies with published studies"). This HC2000_192 cluster, hereafter referred to as S3, could be divided into seven distinct HC1100 clusters (Fig. 4a). One of these S3 subclusters, HC1100_11429, contained exclusively *S. boydii* prov. E1621-54. The other six HC1100 clusters contained two or more *S. flexneri* serotypes per cluster. Connor and coworkers[12] previously subdivided >350 genomes of *S. flexneri* 1-5, X, Y into seven phylogenetic groups (PGs), based on a Bayesian analysis of population structure. As 140 *S. flexneri* genomes from our study were included in the analysis by Connor and coworkers[12], we compared the clustering by cgMLST HC1100 to that obtained by PG. HC1100_204, HC1100_543, HC1100_1468, HC1100_11594, and HC1100_1530 corresponded to PG2, PG4, PG5, PG6 and PG7, respectively (Fig. 4). HC1100_192 encompassed PG1 and PG3, and the use of a higher HC resolution made it possible to link HC400_192 to PG3. However, PG1 did not correspond to a single HC400 cluster. Instead, it corresponded to two such clusters: HC400_237 and HC400_327, which we named PG1a and PG1b, respectively (Fig.4).

Discrepancies with published studies

The SB12 cluster was not identified by Pupo and coworkers[11], or by Yang and coworkers[13]. Instead, the single *S. boydii* 12 strain studied in each of these studies was assigned to their cluster 3 (our cluster S3), in place of *S. boydii* prov. E1621-54 (not included in their studies). As *S. boydii* prov. E1621-54 is serologically related to *S. boydii* 12 (ref. [14]), it seems likely that

their strains were actually *S. boydii* prov. E1621-54 but were mistakenly serotyped as *S. boydii* 12 due to the cross-agglutination between the two serotypes. We also encountered this problem when typing *Shigella* isolates collected from children in Niger[15]. The nine indole-negative isolates obtained, reported to be *S. boydii* 12 at the time, were sequenced and found to belong to HC1100_11429, within cluster S3. Following the use of a typing serum against strain E1621-54, they were reclassified as *S. boydii* prov. E1621-54 (now proposed as *S. boydii* 22, Supplementary Notes section "Updating the *Shigella* typing scheme"). Furthermore, Pupo and coworkers[11] did not report *S. boydii* serotype 11 in both clusters S1 and S2, but only in S2. A genomic study of 117 *Shigella* isolates belonging to validated serotypes found seven *Shigella* clusters following a SNV-based ML phylogeny analysis[5]. The SB12 cluster was not found, and their *S. boydii* 12 strain instead clustered with their cluster 5 (S2 in our study). The analysis of this genome (SRR2994194) with cgMLST and *rfb* cluster analysis revealed that it was actually *S. dysenteriae* 2. Their *S. boydii* 9 strain was also placed in their cluster 11 (our S1), whereas we found this serotype in our S2. The genome they reported (DRR015923) was actually a contamination of *S. boydii* 9 (S1) with *S. boydii* 10 (S2). A recent preprint paper was more comprehensive and used a selection of 485 *Shigella* genomes, selected from over 17,000 publicly available genomes based on ribosomal MLST and ST types[16]. An undescribed phylogeny identified three *Shigella* clusters and seven outliers. The three clusters and five outliers were similar to those from our study. The two remaining, still assigned to *Shigella*, were clusters CSB13 and CSB13-atypical, actually corresponding to *E. albertii*, and attaching and effacing (A/E) *E. coli*, respectively. The status of the provisional serotypes was, however, not addressed adequately, and the continual reporting of different provisional or novel serotypes that are actually identical creates unnecessary confusion for the international surveillance of *Shigella* infections. This issue has now been dealt with by our work (Supplementary Notes section "Updating the *Shigella* typing scheme").

Genomic analysis of metabolic markers used in the current *Shigella* typing scheme

An inability to use/ferment mannitol is a key marker of the *S. dysenteriae* serogroup. However, it was also observed in some strains of *S. boydii* 14, *S. flexneri* 4, and *S. flexneri* 6 (ref. [17]). The mannitol (*mtl*) operon contains three genes: *mtlA* encoding the mannitol permease, *mtlD* encoding the mannitol-1-phosphate dehydrogenase, and *mtlR* encoding the mannitol repressor[18,19]. Different mechanisms of mannitol (*mtl*) operon disruption associated with the mannitol-negative trait have occurred independently in *S. dysenteriae*. In the four different subclusters of S1 containing this serotype, remnants of the *mtlA* and *mtlD* genes, flanked by Insertion Sequences (ISs) IS*1* and IS*3*, are present in S1a genomes; a remnant of *mtlR* disrupted by one IS*1* is present in S1c genomes; and the entire *mtl* operon is lacking in S1d genomes, represented only by *S. dysenteriae* 7. The analysis of a PacBio sequence of *S. dysenteriae* 7 strain ATCC 9052 (ref. [20]), not included in our study, showed a large IS-driven deletion of the entire operon. In the single S2 subcluster containing *S. dysenteriae*, S2d, *mtlR* and a remnant of *mtlD* were present at a chromosomal location different from that in *E. coli* K-12. The mannitol operon was entirely lacking in the three outlier groups of *S. dysenteriae* (SD1, SD8, and SD10).

All strains from the S2 cluster were indole-positive, whereas those from S1 were indole-negative, with one exception: *S. dysenteriae* 7 (indole-positive), assigned to S1d. The loss of indole production has been reported to be due to IS-mediated insertions and/or deletions damaging the tryptophanase (*tna*) operon in a limited number of strains[21]. This operon, which is 3,144 bp long in *E. coli* K-12, contains three genes: *tnaA* encoding a tryptophanase, *tnaB* encoding a permease, and *tnaL* encoding a 25-residue leader peptide[21]. We confirm here that the loss of indole production is associated with the insertion of an IS*1* at base 55 of *tnaL* in the genomes from subclusters S1a, S1b and S1c. In subcluster S1e, containing rare *S. flexneri* 6

genomes, the *tna* alteration occurred independently, but also involved IS*1* integration, this time in the promoter of *tna*. Additional damage (deletions, other IS insertions) was also observed in individual genomes from these subclusters.

Aerogenic strains of *S. boydii* 14 and *S. dysenteriae* 3

By definition, *Shigella* strains do not produce gas from carbohydrate fermentation (except for *S. flexneri* 6), and *S. dysenteriae* strains do not ferment mannitol. The status of aerogenic strains of *S. boydii* 14 long remained a matter of debate. First described in 1943 by Sachs, an aerogenic and mannitol-negative strain referred to as "Enterobacterium A12" (ref.[22]), was considered, by several authors, to be a variant of *S. boydii* 14 (refs. [23–25]), but was considered by Ewing[26,27] to be an *E. coli* O32. We considered two such aerogenic isolates, including the original Sachs strain A12 (CIP 53.44), in the reference dataset. They were grouped with typical *S. boydii* 14 genomes in the S1b subcluster (including, in particular, the reference strain of this serotype, 2770-51). All five recent *S. boydii* 14 isolates from our routine surveillance dataset were also atypical, as they did not ferment mannitol, and three were aerogenic. Another atypical biotype was described for *S. dysenteriae* 3. Two of these aerogenic and mannitol-positive *S. dysenteriae* 3 strains were described in Poland[28] and the UK[14]. We studied the first of these strains (Polska 64-3840). Unlike aerogenic *S. boydii* 14, strain Polska 64-3840 was not placed by cgMLST in the S1a subcluster with other *S. dysenteriae* 3, but in S1b, with *S. flexneri* 6, which is known to produce gas and to ferment mannitol.

Updating the *Shigella* typing scheme

S. dysenteriae prov. 93-119 (ref. [29]), prov. SH-103 (ref. [30]), prov. 204/96 (ref. [31]), prov. 96-3162 (ref. [32]), prov. 97-10607 (ref. [33]), prov. SH-105 (referred to as *S. dysenteriae* 16 by Melito and coworkers[30]) belonged to S1a; *S. boydii* prov. 07-6597 (unpublished) belonged to S1c; *S.*

dysenteriae prov. 96-265 (ref. [20]), prov. BEDP 02-5104 (ref. [30]), and prov. E670/74 (ref. [34]), belonged to S2d; and *S. boydii* prov. E1621-54 (ref. [35]), belonged to HC1100_11429 within S3.

The representative strains of *S. dysenteriae* prov. 204/96 (# 96-204), prov. 96-3162 (# CDC 96-3162), prov. 97-10607 (# 97-10607), and prov. SH-105 (# CDC97026846) had the same biochemical profile[33], and were all agglutinated with the various antisera raised against strains 96-204, 97-10607, SH-105, and KIVI 162. This last strain was not included in this study, but was reported as a new *S. dysenteriae* serotype in Bangladesh[36]. The representative strains of *S. dysenteriae* serotypes prov. 204/96, prov. 96-3162, prov. 97-10607, and prov. SH-105 belonged to HC100_44952 and had identical complete *rfb* clusters of 17·3 kb in size (Fig. 6) and displaying 100% identity (17,306/17,306), with no gaps relative to the *rfb* cluster of *E. albertii* O2 strain SP140152 (GenBank accession code KY574563)[37]. A partial match (91% identity, 7,041/7,744 with three gaps) was also obtained with the *rfb* of *S. dysenteriae* 4 (CP026840). This 17·3 kb *rfb* cluster was not the normal *rfb* cluster (i.e., that located next to the colanic acid biosynthesis gene cluster). This latter consisted only of a remnant of the *S. dysenteriae* 3 *rfb* cluster, damaged by IS*5* (Supplementary Fig. 5). The 17·3 kb *rfb* cluster was located on a ~63 kb genomic island integrated close to the *yqjH* gene (Supplementary Fig. 6). Based on *rfb* sequences, 123 of the 133 HC100_44952 genomes in EnteroBase (6 May 2021) belong to this provisional *S. dysenteriae* serotype, and seven are genuine *S. dysenteriae* 3. The new serotype of *S. dysenteriae* described in the UK as E112707/96 was not included in our study[38]. However, an analysis of its representative genome deposited in EnteroBase (SRR4195641) revealed that it had the same 17·3 kb *rfb* cluster as strain 96-204 and also belonged to HC100_44952. Thus, this novel serotype, described by different groups across the world under different names, is actually identical for all strains considered. It has also become relatively frequent. It ranked first and accounted for 30·2% (16/53) of the *S. dysenteriae* isolates in our French routine

surveillance dataset (2017-2020). Between 2004 and 2017, 19% (150/754) of the *S. dysenteriae* isolates received by Public Health England also belonged to E112707/96 (ref. [38]). We therefore propose its addition to the *Shigella* typing scheme under the name *S. dysenteriae* 16. Strain 96-204 has been deposited in the *Collection de l'Institut Pasteur* (CIP) as the reference strain for this serotype, under CIP 111935.

The representative strains of *S. dysenteriae* prov. 93-119 (# 93-119) and prov. SH-103 (# CDC95011241) had identical biochemical profiles (Supplementary Table 8) and both were agglutinated with an antiserum raised against strain 93-119. They clustered into HC100_35368 and had identical 16·3 kb *rfb* clusters (Fig. 6). This *rfb* cluster displayed no similarity to those of *Shigella* genomes, but was similar (identity:16,307/16,323; gap, 1/16,323) to that of *E. coli* strain YSP-8 (GenBank accession no. CP037910), a non-serotyped *E. coli* collected from pig faeces in China. On the basis of their *rfb* sequences, 17 of the 20 HC100_35368 isolates present in EnteroBase in May 2021 belonged to this new serotype, and three were genuine *S. dysenteriae* 6. We propose adding this new serotype to the *Shigella* serotyping scheme under the name *S. dysenteriae* 17. Strain 93-119 has been deposited in the CIP as the reference strain for this serotype, under CIP 111948.

The three strains from *S. boydii* prov. 07-6597 identified here, associated with travel to Morocco, Chad and Uzbekistan, clustered within S1c close to the genomes of *S. boydii* 1 and 20 (Fig. 2). The three *S. boydii* prov. 07-6597 strains were agglutinated with a serum against strain 07-6597 developed in-house. Their biochemical profile is shown in Supplementary Table 8. The three strains belonged to HC50_45442 and had identical *rfb* clusters of 17·2 kb in size (Fig. 6) that were similar (identities, 17,135/17,260, 99%; gap, 1/17,620) to the *rfb* cluster of *E. coli* O180 strain 86-381 (GenBank accession code AB812077) (Fig. 7). Interestingly, the

first 4 kb of the *S. boydii* prov. 07-6597 *rfb* partially matched that of *S. boydii* 1 (identities 4,230/4,644, 91%; gaps 3/4,644). A search of EnteroBase (6 May 2021) found 16 additional *S. boydii* prov. 07-6597 genomes, all in HC50_45442 (two *S. boydii* 1 genomes were also found in HC50_45442). We therefore propose adding this new serotype to the *Shigella* serotyping scheme under the name *S. boydii* 21. Strain 07-6597 has been deposited in the CIP as the reference strain for this serotype, under CIP 111949.

A non-serotypable *S. boydii* strain (# 07-7164) clustered within S1c close to genomes of *S. boydii* 1 and 20 (Fig. 2). The *rfb* cluster of 07-7164 was similar to that of *S. boydii* 20 (itself derived from that of *S. boydii* 1 via one or two IS*1* insertions, Fig. 6), except that 07-7164 carried an additional IS*1*, inserted within the *wzy* gene and associated with a deletion encompassing the *wbuU* and *wbuW* genes.

S. dysenteriae prov. BEDP 02-5104 (also known as prov. SH-111)[30], and prov. 96-265 clustered together in S2d. Both had two *rfb* clusters: one chromosomal (at the normal site close to the colanic gene cluster) and 10·8 kb in size, and the other plasmid-borne and 11·9 kb in size. The chromosomal cluster was identical to that of *S. dysenteriae* 2, whereas the plasmid-borne cluster was similar (99-100% identities with 11,981/11,981 or 11,978/11,981; no gaps) to the plasmid-borne *rfb* clusters of *E. coli* and *Citrobacter* (GenBank accessions nos. CP048012 and AP022515). These isolates were agglutinated with a serum raised against BEDP 02-5104 but not by the anti-*S. dysenteriae* 2 typing serum, suggesting that expression of the plasmid-borne *rfb* genes has superseded the expression of the chromosomal genes. In EnteroBase, 45 of the 46 genomes with both *rfb* clusters belonged to HC100_11651, which consisted exclusively of these *S. dysenteriae* prov. BEDP 02-5104 genomes (Supplementary Fig. 7). The remaining genome was located in another HC100 cluster, among *S. dysenteriae* 2 genomes. This analysis

suggests that this provisional serotype is actually a *S. dysenteriae* 2 that has acquired an O-antigen-modifying plasmid. In the absence of knowledge about the stability of this plasmid and the possibility of its transfer to *S. dysenteriae* 2 from other HC100 clusters, it seems wise not to consider *S. dysenteriae* prov. BEDP 02-5104 to be a new serotype for the time being.

The representative strain of *S. dysenteriae* prov. E670/74 (# E670/74) was also found in S2d, along with *S. dysenteriae* 2. Its *rfb* cluster was 9·7 kb in size and was similar (99% identities, 9,646/9,709; gaps, 0/9,709) to that of *E. coli* O170 (GenBank accession no. AB812070.1)[39] (Figs. 6 and 7). This provisional serotype was described in 1989 and has not since been reported[34]. We did not identify it in our routine surveillance dataset either. Only four of the 222 S2d genomes in EnteroBase in May 2021 had this 9·7 kb *rfb* cluster, including at least three independent cultures of the same strain, E670/74. Should this serotype be isolated sporadically, it would be considered non-typable in the absence of a dedicated typing serum and genomic sequencing, and would therefore remain undetected. We, therefore, suggest its addition to the *Shigella* serotyping scheme under the name *S. dysenteriae* 18. Strain E670/74 was deposited in the National Collection of Type Cultures (NCTC) as the reference strain for this serotype, under NCTC 11311.

The three *S. boydii* prov. E1621-54 strains studied[14,35], including # E1621-54, belonged to a particular cluster (HC1100_11429) of S3. All the other S3 isolates were *S. flexneri* (serotypes X, Y, and 1-5). These *S. boydii* prov. E1621-54 genomes had an identical 16·9 kb *rfb* cluster, similar (99% identities, 16,879/16,919; gaps, 2/16,919) to that of *E. coli* O7:K1 (GenBank accession no. CP003034) (Figs. 6 and 7). This *rfb* was more distantly related to that of *S. boydii* 12 (Fig. 7). The strains of this provisional serotype – originally described in humans and monkeys from Indonesia – produced indole[14,35]. However, most of our *S. boydii* prov. E1621-

54 isolates were indole-negative. This loss of function was associated with an IS*1* inserted into the promoter region of the *tna* operon (different from the insertion in S1e). One additional *S. boydii* prov. E1621-54 isolate was identified in our routine surveillance dataset. In EnteroBase, approximately 60 non-redundant genomes belonged to HC1100_11429, and all had the 16·9 kb *rfb* cluster of *S. boydii* prov. E1621-54. We therefore propose the addition of this serotype to the *Shigella* typing scheme under the name *S. boydii* 22. Strain E1621-54 has been deposited in the CIP as the reference strain for this serotype, under CIP 111950.

Supplementary Table 1. cgMLST allelic differences between the PacBio and Illumina genomes obtained for identical strains

PacBio genome	Illumina genome	Serotype[1]	cgMLST allelic distances
**54/1621**	E1621-54	SB 22	288
**ATCC 49812**	CIP 57.47	SB 9	67
**ATCC 13313**	CIP 57.28	SD 1	29
**ATCC 49346**	E22383	SD 14	163
**ATCC 49347**	E23507	SD 15	48
**96-3162**	96-3162	SD 16	365
**204/96**	96-204	SD 16	9
**E670/74**	E670/74	SD 18	24
**ATCC 9754**	CIP 52.32	SD 6	584
**ATCC 12037**	CIP 58.26	SD 9	12
**74-1170**	NCDC1170-74	SF 5a	16

[1]SB, *S. boydii*; SD, *S. dysenteriae*; SF, *S. flexneri*

Supplementary Table 2. GenBank accession numbers of the *Shigella* O-antigen gene clusters

O-antigen gene cluster from:	Accession number
S. dysenteriae 1	MZ286368
S. dysenteriae 2	MZ286369
S. dysenteriae 3	MZ286370
S. dysenteriae 4	MZ286371
S. dysenteriae 5	MZ286372
S. dysenteriae 6	MZ286373
S. dysenteriae 7	MZ286374
S. dysenteriae 8	MZ286375
S. dysenteriae 9	MZ286376
S. dysenteriae 10	MZ286364
S. dysenteriae 11	MZ286365
S. dysenteriae 12	MZ286366
S. dysenteriae 13	MZ286367
S. dysenteriae 14	MF322749
S. dysenteriae 15	MF322748
S. dysenteriae 16 (prov. 204/96)	MF322751
S. dysenteriae 17 (prov. 93-119)	MF322752
S. dysenteriae 18 (prov. E670/74)	MF322750
S. dysenteriae prov. BEDP 02-5104	MZ303046
S. boydii 1	MZ286385
S. boydii 2	MZ286387
S. boydii 3	MZ286388
S. boydii 4	MZ286389
S. boydii 5	MZ286390
S. boydii 6	AF402314
S. boydii 7	MZ286391
S. boydii 8	MZ286392
S. boydii 9	MZ286393
S. boydii 10	MZ286378
S. boydii 11	MZ286379
S. boydii 12	EU296406
S. boydii 14	MZ286380
S. boydii 15	MZ286381
S. boydii 16	MZ286382
S. boydii 17	MZ286383
S. boydii 18	MZ286384
S. boydii 19	MF322754
S. boydii 20	MZ286386
S. boydii 21 (prov. 07-6597)	MF322754
S. boydii 22 (prov. E1621-54)	MF322747
S. flexneri 1-5, X, Y	MZ286377
S. flexneri 6	MZ286394
S. sonnei (plasmid)	AF285971
S. sonnei (chromosome)	AF031957

Supplementary Table 3. GenBank accession numbers and coordinates of the gene and genetic structures studied

Target	Strain	Accession no.	Coordinates
mtlA	*Escherichia coli* K-12 substr. MG1655	NC_000913.3	3772281-3774194
mtlD	*Escherichia coli* K-12 substr. MG1655	NC_000913.3	3774424-3775572
mtlR	*Escherichia coli* K-12 substr. MG1655	NC_000913.3	3775572-3776159
tnaC	*Escherichia coli* K-12 substr. MG1655	NC_000913.3	3888435-3888509
tnaA	*Escherichia coli* K-12 substr. MG1655	NC_000913.3	3888730-3890145
tnaB	*Escherichia coli* K-12 substr. MG1655	NC_000913.3	3890236-3891483
ipaH	*Shigella boydii* CDC 3083-94	CP001063.1	1918645-1920360
rafABD	*Escherichia coli* K-12	M27273.1	1-5284
rafY	*Escherichia coli*	U82290.1	1-1866

Supplementary Table 4. Number of *Shigella* strains and isolates studied per dataset

Serotype	Reference	Reference+	Routine
S. boydii	**70**	**10**	**101**
1	3	0	5
2	3	0	40
3	3	0	0
4	5	1	10
5	4	2	2
6	1	0	0
7	3	0	0
8	3	0	2
9	4	3	1
10	4	0	7
11	5	0	14
12	2	2	0
14	6	0	5
15	2	0	0
16	2	0	0
17	1	1	0
18	3	1	4
19	3	0	5
20	6	0	5
21	3	0	0
22	3	0	1
Rough	0	0	0
NST	1	0	0
S. dysenteriae	**81**	**2**	**53**
1	16	0	0
2	7	0	14
3	3	0	7
4	4	0	1
5	3	0	0
6	2	0	0
7	3	0	0
8	3	2	0
9	3	0	1
10	2	0	0
11	2	0	0
12	4	0	7
13	2	0	0
14	1	0	0
15	1	0	0
16	6	0	16
17	6	0	1
18	1	0	0
prov. BEDP 02-5104	12	0	2
Rough	0	0	3
NST	0	0	1
S. sonnei	**44**	**0**	**2161**
Lineage I	11	0	14
Lineage II	15	0	108
Lineage III	17	0	2039
Lineage IV	1	0	0
***S. flexneri* 1-5**	**103**	**65**	**1423**
**Lineage I**	**32**	**12**	**460**
1a	6	1	9
1b	9	0	338
1c/7a	2	3	74
7b	3	0	11

2a	0	0	1
2b	2	2	1
3b	3	0	8
4a	0	2	1
4av	2	3	12
4b	3	1	0
4bv	0	0	0
X	1	0	1
Y	1	0	1
Yv	0	0	3
**Lineage II**	**25**	**2**	**218**
3a	18	2	200
3b	7	0	16
Unknown	0	0	1
X	0	0	0
Y	0	0	1
**Lineage III**	**31**	**10**	**680**
1a	1	1	1
2	0	0	2
2a	19	0	599
2b	1	5	40
4	0	0	0
5a	2	0	0
X	1	0	2
Xv	4	0	20
Y	1	4	14
Yv	2	0	1
Rough	0	0	1
**Lineage IV**	**9**	**9**	**11**
3a	5	1	11
3b	1	2	0
4bv	1	1	0
X	2	1	0
NST	0	4	0
**Lineage V**	**2**	**8**	**0**
5a	2	4	0
5b	0	4	0
**Lineage VI**	**2**	**14**	**3**
Y	2	5	0
Yv	0	7	3
Xv	0	2	0
**Lineage VII**	**2**	**10**	**51**
4	0	0	1
4a	0	1	12
4av	1	3	31
Y	0	5	0
Yv	1	1	7
***S. flexneri* 6**	**19**	**4**	**132**
Boyd 88	12	0	120
Hertfordshire	4	0	9
Manchester	2	0	3
Newcastle	1	0	0
Unknown	0	4	0
**TOTAL**	**317**	**81**	**3870**

NST, non-serotypable

Supplementary Table 5: cgMLST allelic differences observed between pairs of isolates from the same patient

Isolate # 1	Isolate # 2	Serotype[1]	cgMLST allelic difference	Time interval between samples, in days
201700699	201700700	SD 2	0	0
201701574	201701177	SF 2a	4	41
201702189	201702190	SF 2a	1	0
201702422	201702619	SON	1	11
201702425	201702482	SON	1	0
201702489	201702493	SON	0	9
201702491	201702785	SON	0	3
201702492	201702518	SON	2	0
201704164	201704239	SF 1b	3	0
201704289	201704335	SON	3	5
201704554	201705129	SON	2	19
201705047	201705059	SF 1b	3	4
201706769	201706374	SF 2a	1	3
201707086	201707593	SON	3	12
201707285	201707469	SON	3	3
201708044	201708196	SON	0	4
201708831	201709932	SD 12	0	7
201709780	201711502	SON	3	60
201801253	201801603	SON	3	16
201803392	201803594	SF 1b	0	3
201805339	201805383	SF 1b	0	6
201807341	201808646	SF 6	0	11
201807678	201807018	SON	1	10
201808860	201809690	SON	1	28
201810618	201810845	SON	1	5
201901699	201902697	SF 2a	5	27
201902285	201901958	SF 7b	2	3
201902507	201902449	SF 2a	2	11
201903629	201903788	SF 6	2	6
201903789	201903630	SF 6	0	8
201910698	201910787	SON	0	1
201911113	201910946	SF 2a	1	7
201911370	201908021	SF 1b	3	90
202008533	202008379	SON	1	7

SD, *S. dysenteriae*; SF, *S. flexneri*; SON, *S. sonnei*.

Supplementary Table 6. *In silico* serotype prediction for 316 *Shigella* reference strains with serotype designation, obtained with various tools

		% assignment:															
		**EnteroBase SeroPred**				**ShigaTyper**				**ShigEiFinder (Fasta)**				**ShigEiFinder (Reads)**			
**Serotype**	*n*	C	U	I	N	C	U	I	N[#]	C	U	I	N	C	U	I	N
S. boydii																	
**1**	3	0	100 (B1/O149)			100				100				100			
**2**	3	100				67			33	100				100			
**3**	3	100				100				100				100			
**4**	5	20	80 (B4/O53)			80			20	100				100			
**5**	4	100				50			50	100				100			
**6**	1	100							100	100				100			
**7**	3	100				100				100				100			
**8**	3	100				100				100				100			
**9**	4	100				100				100				100			
**10**	4			100 (B6)					100			100 (B6)				100 (B6)	
**11**	5	60		20 (O105)	20	60		20 (Not)	20	80	20 (Cs1)			60	20 (Cs1)	20[!]	
**12**	2	100				100				100				100			
**14**	6	100				100				83	17 (Cs1)			100			
**15**	2		100 (B15/O112)			100				100				100			
**16**	2	100				100				100				100			
**17**	1	100				100				100				100			
**18**	3	100				100				100				100			
**19**	3				100	67			33	100				100			
**20**	6			100 (B1/O149)		67			33	100				100			
**21***	3			100 (O180)				67 (B18)	33		100 (Cs1/O180)				100 (Cs1/O180)		
**22***	3			100 (O7)		100				100				100			
S. dysenteriae																	
**1**	16	100				100				100				100			
**2**	7	86			14	100				86	14 (Cs2)			86	14 (Cs2)		
**3**	3			100 (O124, O164)		100				100				100			
**4**	4			75 (O168/OX6)	25	100				100				75	25 (Cs1)		
**5**	3			100 (O58)		100				100				100			
**6**	2			100 (O130)		100				100				100			

**7**	3			100 (O121)		100				100				100			
**8**	3			100 (O38)		100				100				100			
**9**	3			100 (O40)		100				100				100			
**10**	2	50			50	100				100				100			
**11**	2		50 (D11/O29)	50 (O29)		100				100				100			
**12**	4			100 (O152)		100				100				100			
**13**	2			100 (O150)		100				100				100			
**14**	1				100	100				100				100			
**15**	1				100	100				100				100			
**16***	6				100			67 (p. 96-265)	33		17 (Cs1)	83 (p. 96-265)			17 (Cs1)	83 (p. 96-265)	
**17***	6				100				100		100 (Cs1)				100 (Cs1)		
**18***	1			100 (O170)		100				100				100			
**p. 02-5104**	12			100 (D2)				100 (D2)				100 (D2)				100 (D2)	
S. flexneri																	
**1-5, X, Y**	103	58	36 (F1-5/O13)	1 (O129/O13)	5	100				100				100			
**1a**	7				100	100				100				100			
**1b**	9				100	100				100				100			
**1c**	2				100	100					100 (1c/7b)				100 (1c/7b)		
**2a**	19				100	100				100				100			
**2b**	3				100	100				100				100			
**3a**	23				100	100				100				100			
**3b**	11				100	73	18 (new)	9 (1b)		73	18[¥]	9 (1b)		73	18[¥]	9 (1b)	
**4av**	3				100	67		33 (4bv)		67	33 (4av/4b)			67	33 (4av/4b)		
**4b**	3				100	100				100				100			
**4bv**	1				100	100					100 (4av/4b)				100 (4av/4b)		
**5a**	4				100	100				100				100			
**7b**	3				100	100					100[¥]				100[¥]		
**X**	4				100	50		50 (3a, Xv)		75		25 (3a)		75		25 (3a)	
**Xv**	4				100	100				100				100			
**Y**	4				100	50		50 (X)		50		50 (X)		50		50 (X)	
**Yv**	3				100	67		33 (Xv)		100				100			
**6**	19	79		21 (O147)		95			5	95		5[!]		100			
S. sonnei	44				100	75			25	95		5[!]		97.7		2.3[!]	

C, correct; U, uncertain; I, incorrect; N, none; *, novel *Shigella* serotype described in our study; [#], no prediction for ShigaTyper (no *wzx*, multiple *wzx* or error); new, novel serotype; Not, not *Shigella* or EIEC; [!], *Shigella* or EIEC unclustered; B, *S. boydii*; D, *S. dysenteriae*; F, *S. flexneri*; p. 96-265, *S. dysenteriae* prov. 96-265; Cs1, Cluster 1 (*Shigella*) from ref. [16] ; Cs2, Cluster 2 (*Shigella*) from ref. [16]; [¥], Cluster 3 (*S. flexneri*) from ref. [16].

Supplementary Table 7. *In silico* serotype prediction for 3,861 *Shigella* routine isolates with serotype designation, obtained with various tools

		% assignment:															
		EnteroBase				ShigaTyper				ShigEiFinder (Fasta)				ShigEiFinder (Reads)			
**Serotype**	*n*	**C**	**U**	**I**	**N**	**C**	**U**	**I**	**N**[#]	**C**	**U**	**I**	**N**	**C**	**U**	**I**	**N**
S. boydii																	
**1**	5		20 (B1/O149)		80	60		20 (EIEC)	20	40	60[ǂ]				20[ǂ]	80[!]	
**2**	40	2.5			97.5	92.5			7.5	75	22.5[ǂ]	2.5 (EIEC)			47.5[ǂ]	50[!] 2.5 (EIEC)	
**4**	10	10			90	90			10	80	20[ǂ]				70[ǂ]	30[!]	
**5**	2				100	100				100					100 (Cs2)		
**8**	2				100	100					100[ǂ]				50[ǂ]	50[!]	
**9**	1				100	100				100					100 (Cs2)		
**10**	7		14.3[+]		85.7	0			100			100 (B6)			85.7[ǂ]	14.3[!]	
**11**	14			14.3 (O105)	85.7	86			14	78.5	21.5[ǂ]				43[ǂ] 7 (O105/Cs1)	50[!]	
**14**	5				100	100				100					40[ǂ]	60[!]	
**18**	4				100	100				75	25[ǂ]				100[ǂ]		
**19**	5				100	100				80	20[ǂ]			20	60[ǂ]	20[!]	
**20**	5			20 (B1/O149)	80	0		100 (B1)		100					80[ǂ]	20[!]	
**22***	1				100	100				100					100[¥]		
S. dysenteriae																	
**2**	14	14.3	7.1[+]		78.6	92.8			7.2	92.8	7.2 (Cs2)				100 (Cs2)		
**3**	7	14.3			85.7	85.7		14.3 (EIEC)		71.4	14.3[ǂ] 14.3 (D3/Cs3, CSS)			14.3	28.6[ǂ]	57.1[!]	
**4**	1				100	100					100[ǂ]					100[!]	
**9**	1	100				100				100				100			
**12**	7	14.3			85.7	100				85.7	14.3[ǂ]				14.3	85.7	
**16**	16				100	0		75 (p. 96-265)	25		75[ǂ]	18.75 (p. 96-265) 6.25 (F6)			31.25[ǂ]	68.75[!]	
**17***	1				100	0			100		100[ǂ]					100[!]	
**p. 02-5104**	2				100	0		100 (D2)				100 (D2)			50 (Cs2)	50[!]	
S. flexneri																	
**1-5,X,Y**	1418	7.6	2.9[+]	0.4 (O13)	89.1	99		0.6 (EIEC)	0.4	93.4	6.4[¥]	0.2[!]		0.1	99.7[¥]	0.2[!]	
**1a**	10				100	40		50 (1b)		30	50[¥]	20 (1b)			100[¥]		

								10 (1C)									
**1b**	338				100	99.4		0.6 (EIEC)		63.7	36.3$^{¥}$				100$^{¥}$		
**1c**	74				100	100					63.5 (1c/7b)<br>36.5$^{¥}$				100$^{¥}$		
**2a**	600				100	97.5	0.3	1 (Y)<br>0.8 (EIEC)<br>0.1 (new)	0.3	56.7	42.4$^{¥}$<br>0.2 (O13/O129/ O135, F2a, SS)	0.5 (Y)<br>0.2$^{!}$		0.2	99.5$^{¥}$	0.3$^{!}$	
**2b**	41				100	90.2		9.8 (2a)		46.3	46.3$^{¥}$	7.4 (2a)			100$^{¥}$		
**3a**	211				100	95.7		2.8 (3b)<br>0.5 (EIEC)	1	59.5	38$^{¥}$	2 (3b)<br>0.5$^{!}$		0.5	99.5$^{¥}$		
**3b**	24				100	54		29 (new)<br>8 (3a)<br>4 (1b)	5	58.5	37.5$^{¥}$	4 (3a)			100$^{¥}$		
**4a**	13				100	7.7		84.6 (4av)<br>7.7 (EIEC)		7.7	23.1$^{¥}$	69.2 (4av)			100$^{¥}$		
**4av**	43				100	100				55.8	44.2$^{¥}$				100$^{¥}$		
**7b**	11				100	81.8		9.1 (EIEC)	9.1	9.1	45.5 (1c/7b)<br>45.4$^{¥}$				100$^{¥}$		
**X**	3				100	33.4		33.3 (X)<br>33.3 (new)			100$^{¥}$				100$^{¥}$		
**Xv**	20				100	90		5 (Yv)<br>5 (new)		45	45$^{¥}$	5 (2a)<br>5 (Yv)			100$^{¥}$		
**Y**	16				100	75		18.75 (2a)<br>6.25 (3b)		68.8	18.8$^{¥}$	6.2 (2a)<br>6.2 (3b)			100$^{¥}$		
**Yv**	14				100	43		28.5 (4av)<br>28.5 (Xv)		14.3	50$^{¥}$	21.4 (4av)<br>14.3 (Xv)			100$^{¥}$		
**6**	132	10.6	7.6^{+}		81.8	96.2		0.8 (EIEC)	3	86.3	13.7^{ǂ}			3.8	47^{ǂ}	49.2$^{!}$	
S. sonnei	2161	0.8			99.2	98.8		0.3 (EIEC)<br>0.1 (Not)	0.8	99.1		0.9$^{!}$		99.4		0.6$^{!}$	

C, correct; U, uncertain; I, incorrect; N, none; *, novel *Shigella* serotype described in our study; #, no prediction for ShigaTyper (no *wzx*, multiple *wzx* or error); new, novel serotype; Not, not *Shigella* or EIEC; !, *Shigella* or EIEC unclustered; +, "uncertain" according to SeroPred; B, *S. boydii*; D, *S. dysenteriae*; F, *S. flexneri*; p. 96-265, *S. dysenteriae* prov. 96-265; Cs1, Cluster 1 (*Shigella*) from ref. [16]; Cs2, Cluster 2 (*Shigella*) from ref. [16]; ǂ, 0:H45/Cluster 1 from ref. [16]; CSS, *S. sonnei* cluster from ref. [16]; ¥, Cluster 3 (*S. flexneri*) from ref. [16].

Supplementary Table 8. Biochemical characteristics of new serotypes *S. dysenteriae* 17 and *S. boydi* 21.

**Test**	*S. dysenteriae* 17	*S. boydii* 21
Motility	-	-
Oxidase	-	-
β-galactosidase (ONPG)	-	-
Lysine decarboxylase	-	-
Ornithine decarboxylase	-	-
Arginine dihydrolase	-	-
Tryptophan deaminase	-	-
Indole production	-	-
Voges-Proskauer (37°C)	-	-
Urea hydrolysis	-	-
H_2S production	-	-
Gelatinase	-	-
NO_2 from NO_3	+	+
Citrate utilisation	-	-
Glucose (gas)	-	-
Acid from:		
D-adonitol	-	-
Amidon	-	-
L-arabinose	-[a]	+
D-cellobiose	-	-
Dulcitol	-	-
D-fructose	+	+
L-fucose	-	-
D-galactose	+	+
D-glucose	+	+
Glycerol	(+)	(+)
Inositol	-	-
D-lactose	-	-
D-maltose	-	-
D-mannitol	-	+
D-mannose	+	+
N-acetyl glucosamine	+	+
Potassium 2-keto-gluconate	-	-
Potassium 5-keto-gluconate	-	-
Potassium gluconate	+	+
D-raffinose	-	-
L-rhamnose	-	-
L-ribose	+	+
Salicin	-	-
D-sorbitol	-	-
L-sorbose	(+)	-
D-sucrose	-	-
D-trehalose	+	+
D-xylose	-	-
L-xylose	-	-

+, all strains positive (one day); -, all strains negative; (+), all strains positive (two days);
[a], positive in one day with the API 50 CH strip (negative with the API 20 E strip).

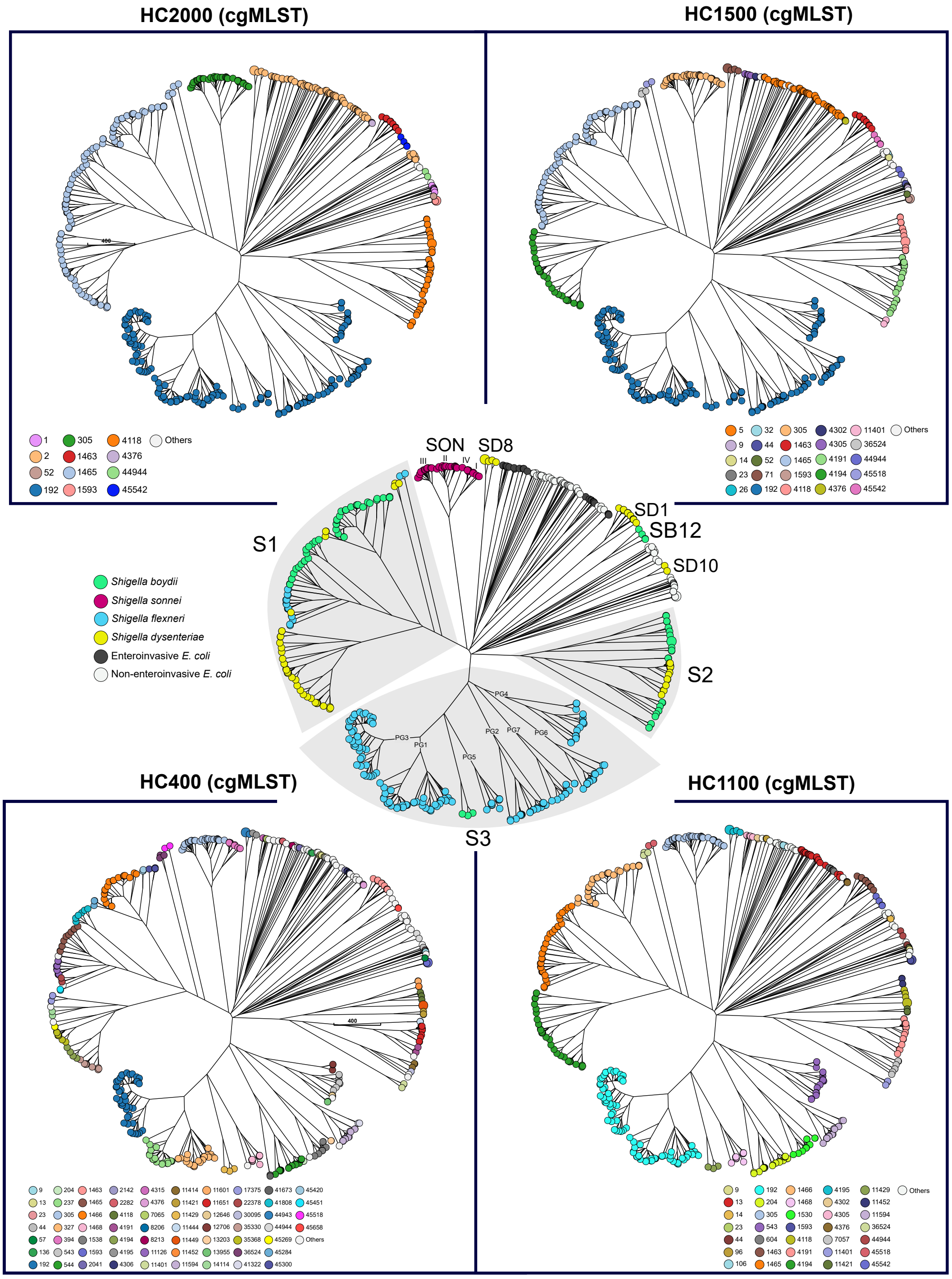

Supplementary Figure 1: A NINJA neighbour-joining GrapeTree showing the population structure of *Shigella* spp. based on the cgMLST allelic differences between 493 *Shigella* and *E. coli* reference genomes. Central Tree: the tree nodes are colour-coded by *Shigella* serogroup and *E. coli* pathovar. The different *Shigella* cgMLST clusters are labelled. For the SON cluster, the different genomic lineages of *S. sonnei* are indicated with Latin numerals. For the *S. flexneri* serotypes in cluster S3, the phylogenetic groups (PG1 to PG7) are also indicated. Peripheral trees: the tree nodes are colour-coded by different cgMLST HCs data (from HC400 to HC2000).

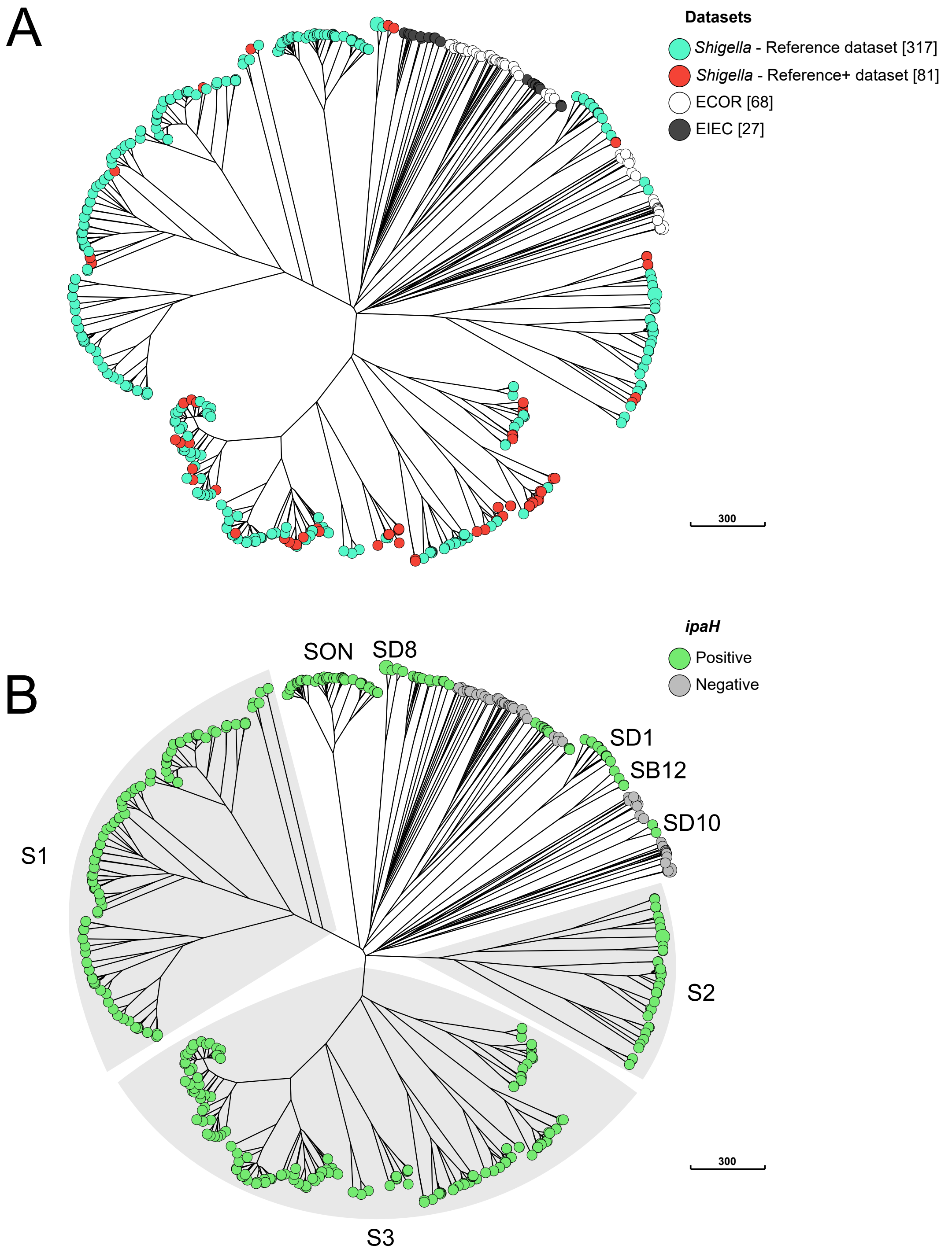

Supplementary Figure 2. A NINJA neighbour-joining GrapeTree showing the population structure of *Shigella* spp. based on the cgMLST allelic differences between 493 *Shigella* and *E. coli* reference genomes. This tree is the same as that shown in Figure 1. (A) The tree nodes are colour-coded by type of dataset, to illustrate the differential contribution to *Shigella* population diversity of the reference and reference+ datasets. (B) The tree nodes are colour-coded according to the presence or absence of the *ipaH* gene. The numbers of isolates in each dataset are indicated in brackets.

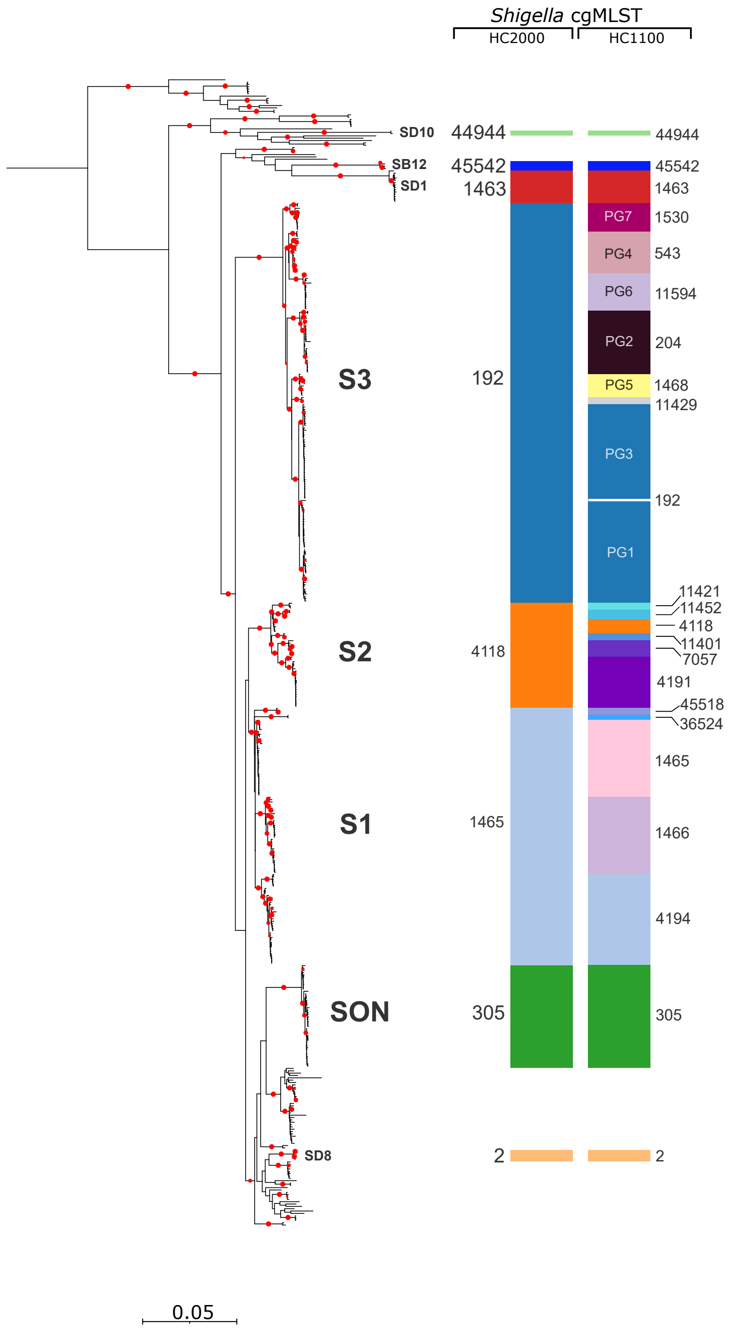

Supplementary Figure 3. A maximum-likelihood phylogenetic tree showing the population structure of 491 *Shigella* and *E. coli* reference genomes based on 5,129 recombination-filtered core-genome single-nucleotide variants (SNVs). Nodes supported by bootstrap values ≥95% are indicated by red dots. Phylogenetic clades containing *Shigella* genomes are labelled with the same nomenclature (S1-S3, SON, SD1, SD8, SD10, and SB12) as in Figure 1. All the *Shigella* genomes are also labelled on the right with cgMLST HC2000 and HC1100 data. Two genomes (*E. fergusonii* RHB19-C05 and *S. dysenteriae* type 1 CAR7) were removed from the analysis by the Gubbins software.

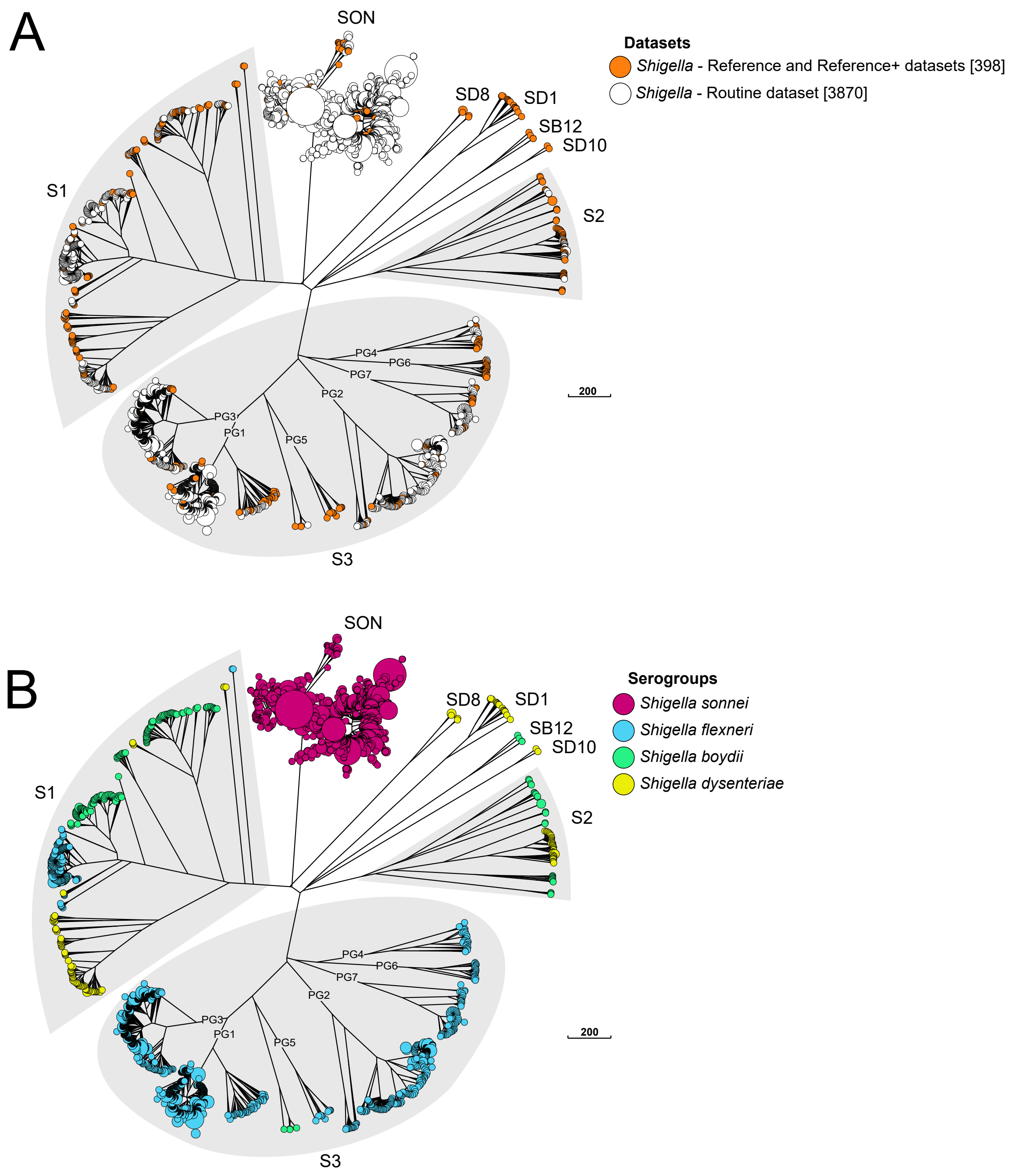

Supplementary Figure 4. A NINJA neighbour-joining GrapeTree showing the genomic diversity of 4,268 *Shigella* isolates, based on the "reference" (*n* = 317), "reference+" (*n* = 81), and "routine" (*n* = 3,870) datasets. The tree nodes are colour-coded by type of dataset (A) or by *Shigella* serogroup (B). The global dataset consists of the reference and reference+ datasets. The numbers of isolates in each dataset are indicated in brackets.

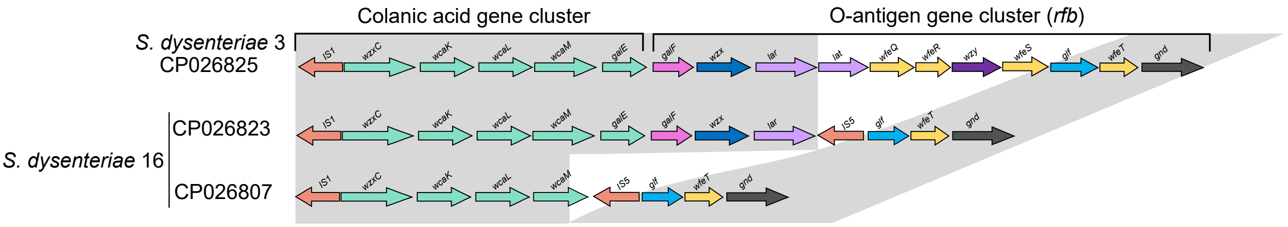

Supplementary Figure 5: Representation of the normal O-antigen gene cluster locus in *S. dysenteriae* 3 and *S. dysenteriae* 16 isolates.

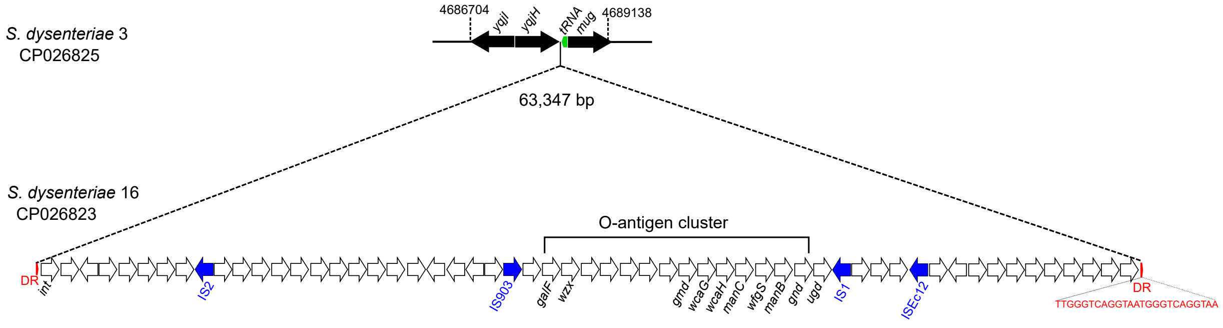

Supplementary Figure 6: Representation of the genomic island containing the complete O-antigen cluster in *S. dysenteriae* 16.

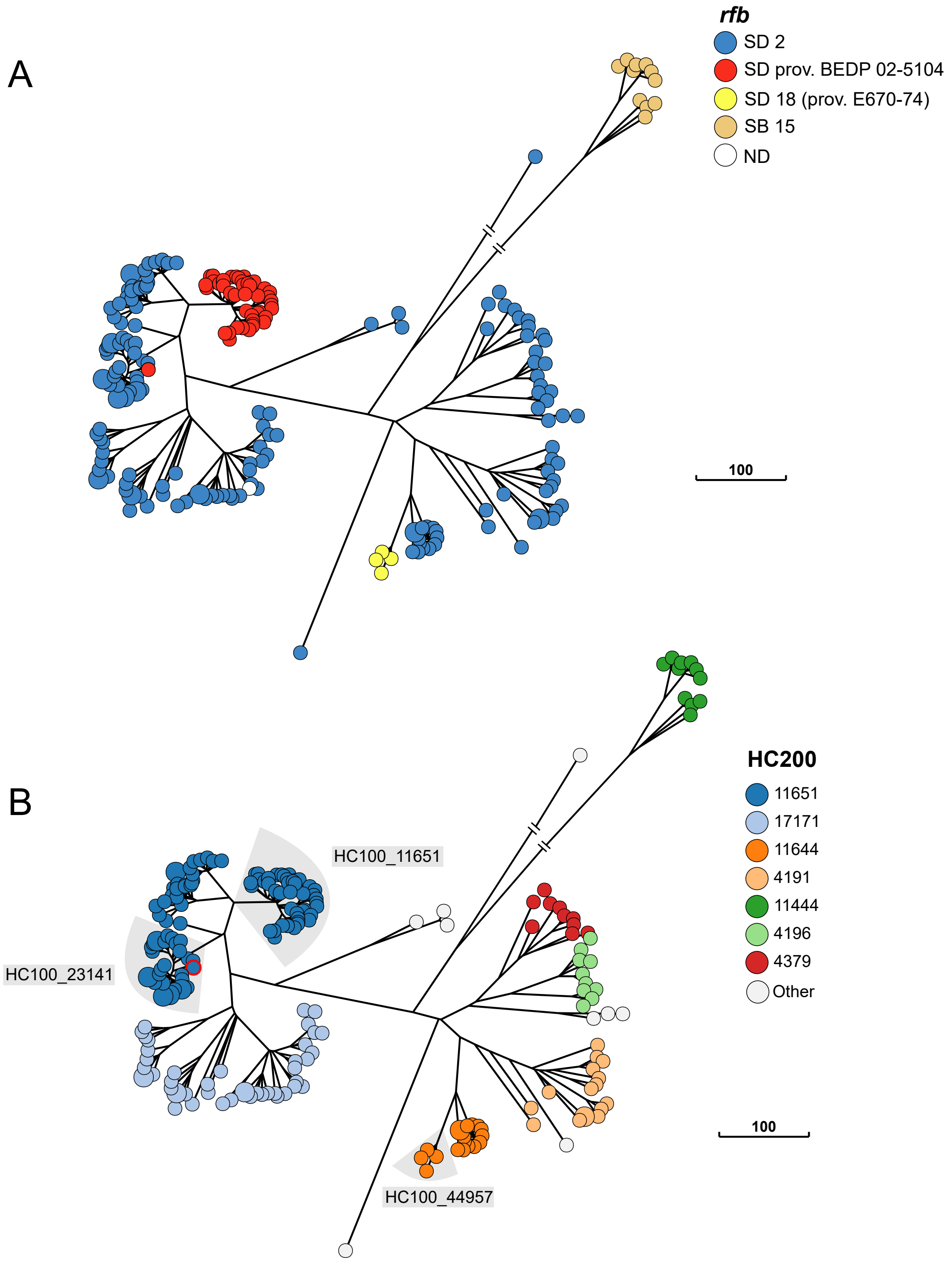

Supplementary Figure 7: NINJA neighbour-joining GrapeTree based on the cgMLST data for all S2d genomes (HC1100_4191, n = 222) present in EnteroBase (May 06 2021). (A) The tree nodes are colour-coded by type of *rfb* gene. ND indicates that the *rfb* cluster could not be identified. (B) The tree nodes are colour-coded by HC200 data. HC200 groups with fewer than three isolates are represented by white nodes. All *S. dysenteriae* prov. BEDP 02-5104 fall within HC100_11651, except for one strain (highlighted in red).

Supplementary References

1. Zhou, Z. *et al.* The EnteroBase user's guide, with case studies on *Salmonella* transmissions, *Yersinia pestis* phylogeny, and *Escherichia* core genomic diversity. *Genome Res.* **30**, 138–152 (2020).
2. Lan, R., Alles, M. C., Donohoe, K., Martinez, M. B. & Reeves, P. R. Molecular evolutionary relationships of enteroinvasive *Escherichia coli* and *Shigella* spp. *Infect. Immun.* **72**, 5080–5088 (2004).
3. Hazen, T. H. *et al.* Investigating the relatedness of enteroinvasive *Escherichia coli* to other *E. coli* and *Shigella* isolates by using comparative genomics. *Infect. Immun.* **84**, 2362–2371 (2016).
4. Dhakal, R., Wang, Q., Howard, P. & Sintchenko, V. Genome sequences of enteroinvasive *Escherichia coli* sequence type 6, 99, and 311 strains acquired in Asia Pacific. *Microbiol. Resour. Announc.* **8**, e00944-19 (2019).
5. Pettengill, E. A., Pettengill, J. B. & Binet, R. Phylogenetic analyses of *Shigella* and enteroinvasive *Escherichia coli* for the identification of molecular epidemiological markers: whole-genome comparative analysis does not support distinct genera designation. *Front. Microbiol.* **6**, 1573 (2015).
6. Ochman, H. & Selander, R. K. Standard reference strains of *Escherichia coli* from natural populations. *J. Bacteriol.* **157**, 690–693 (1984).
7. Galardini, M. *et al.* Phenotype inference in an *Escherichia coli* strain panel. *Elife* **6**, e31035 (2017).
8. Clermont, O., Gordon, D. & Denamur, E. Guide to the various phylogenetic classification schemes for *Escherichia coli* and the correspondence among schemes. *Microbiology* **161**, 980–988 (2015).

9. Coimbra, R. S., Grimont, F. & Grimont, P. A. Identification of *Shigella* serotypes by restriction of amplified O-antigen gene cluster. *Res. Microbiol.* **150**, 543–553 (1999).
10. Hyma, K. E. *et al.* Evolutionary genetics of a new pathogenic *Escherichia* species: *Escherichia albertii* and related *Shigella boydii* strains. *J. Bacteriol.* **187**, 619–628 (2005).
11. Pupo, G. M., Lan, R. & Reeves, P. R. Multiple independent origins of *Shigella* clones of *Escherichia coli* and convergent evolution of many of their characteristics. *Proc. Natl. Acad. Sci. U. S. A.* **97**, 10567–10572 (2000).
12. Connor, T. R. *et al.* Species-wide whole genome sequencing reveals historical global spread and recent local persistence in *Shigella flexneri*. *Elife* **4**, e07335 (2015).
13. Yang, J. *et al.* Revisiting the molecular evolutionary history of *Shigella* spp. *J. Mol. Evol.* **64**, 71–79 (2007).
14. Ewing, W. H., Reavis, R. W. & Davis, B. R. Provisional *Shigella* serotypes. *Can. J. Microbiol.* **4**, 89–107 (1958).
15. Langendorf, C. *et al.* Enteric bacterial pathogens in children with diarrhea in Niger: diversity and antimicrobial resistance. *PLoS One* **10**, e0120275 (2015).
16. Zhang, X., Payne, M., Nguyen, T., Kaur, S. & Lan, R. Cluster-specific gene markers enhance *Shigella* and Enteroinvasive *Escherichia coli in silico* serotyping. *bioRxiv* (2021) doi:10.1101/2021.01.30.428723.
17. Edwards, P. R. & Ewing, W. H. Genus *Shigella*. in Identification of *Enterobacteriaceae* 108–142 (Burgess Publishing Company, 1972).
18. Davis, T., Yamada, M., Elgort, M. & Saier, M. H. Nucleotide sequence of the mannitol (*mtl*) operon in *Escherichia coli*. *Mol. Microbiol.* **2**, 405–412 (1988).
19. Figge, R. M., Ramseier, T. M. & Saier, M. H. The mannitol repressor (MtlR) of *Escherichia coli*. *J. Bacteriol.* **176**, 840–847 (1994).

20. Kim, J. *et al.* High-quality whole-genome sequences for 59 historical *Shigella* strains generated with PacBio sequencing. *Genome Announc.* **6**, e00282-18 (2018).

21. Rezwan, F., Lan, R. & Reeves, P. R. Molecular basis of the indole-negative reaction in *Shigella* strains: extensive damages to the *tna* operon by insertion sequences. *J. Bacteriol.* **186**, 7460–7465 (2004).

22. Sachs, H. A report on an investigation into the characteristics of new types of non-mannitol-fermenting bacilli isolated from cases of bacillary dysentery in India and Egypt. *J. R. Army. Med. Corps.* (1943) doi:10.1136/jramc-80-02-07.

23. Carpenter, K. P. The relationship of the enterobacterium A 12 (Sachs) to *Shigella boydii* 14. *J. Gen. Microbiol.* **26**, 535–542 (1961).

24. Regina, M., Toledo, F., Silva, R. M. & Trabulsi, L. R. Sachs' "Enterobacterium A12" is an aerogenic Variant of *Shigella boydii* 14. *Int. J. Syst. Evol.* **31**, 242–244 (1981).

25. Szturm-Rubinstein, S., Piechaud, D. & Allos, G. A propos d'une variété gazogène de *Shigella boydii* 14. *Ann. Inst. Pasteur (Paris)* **103**, 303–305 (1962).

26. Edwards, P. R. & Ewing, W. H. Edwards and Ewing's identification of *Enterobacteriaceae*. (Elsevier Publishing Co., New York, N.Y, 1986).

27. Ewing, W. H. & Hucks, M. C. Two intermediate members of *Enterobacteriaceae*. *J. Bacteriol.* **60**, 367–368 (1950).

28. Stypulkowska, H. Atypical strain of *Shigella dysenteriae* 3 isolated in Poland. *Med. Dosw. Mikrobiol.* **16**, 147–154 (1964).

29. Matsushita, S. *et al.* *Shigella dysenteriae* strains possessing a new serovar isolated from imported diarrheal cases in Japan. *Kansenshogaku Zasshi* **71**, 412–416 (1997).

30. Melito, P. L. *et al.* A novel *Shigella dysenteriae* serovar isolated in Canada. *J. Clin. Microbiol.* **43**, 740–744 (2005).

31. Matsushita, S. *et al.* *Shigella dysenteriae* strains possessing a new serovar (204/96) isolated from imported diarrheal cases in Japan. *Kansenshogaku Zasshi* **72**, 499–503 (1998).

32. Kuijper, E. J., van Eeden, A., de Wever, B., van Ketel, R. & Dankert, J. Nonserotypeable *Shigella dysenteriae* isolated from a Dutch patient returning from India. *Eur. J. Clin. Microbiol. Infect. Dis.* **16**, 553–554 (1997).

33. Coimbra, R. S. *et al.* Molecular and phenotypic characterization of potentially New *Shigella dysenteriae* serotype. *J. Clin. Microbiol.* **39**, 618–621 (2001).

34. Gross, R. J., Thomas, L. V., Cheasty, T., Rowe, B. & Lindberg, A. A. Four new provisional serovars of *Shigella*. *J. Clin. Microbiol.* **27**, 829–831 (1989).

35. Takasaka, M. *et al.* Isolation and pathogenicity of provisional serovar 1621-54 of '*Shigella*' from imported cynomolgus monkeys. *Jpn. J. Med. Sci. Biol.* **36**, 27–37 (1983).

36. Talukder, K. A. *et al.* A novel serovar of *Shigella dysenteriae* from patients with diarrhoea in Bangladesh. *J. Med. Microbiol.* **56**, 654–658 (2007).

37. Ooka, T. *et al.* O-antigen biosynthesis gene clusters of *Escherichia albertii*: their diversity and similarity to *Escherichia coli* gene clusters and the development of an O-genotyping method. *Microb. Genom.* **5**, e000314 (2019).

38. Terry, L. M. *et al.* Antimicrobial resistance profiles of *Shigella dysenteriae* isolated from travellers returning to the UK, 2004-2017. *J. Med. Microbiol.* **67**, 1022–1030 (2018).

39. Iguchi, A. *et al.* A complete view of the genetic diversity of the *Escherichia coli* O-antigen biosynthesis gene cluster. *DNA Res.* **22**, 101–107 (2015).
