# Supplementary material for: Population structure analysis and laboratory monitoring of Shigella by core-genome multilocus sequence typing
Source: Nat Commun. 2022 Jan 27;13:551. doi: 10.1038/s41467-022-28121-1 (PMC8795385; doi:10.1038/s41467-022-28121-1)
Supplement: Supplementary file 2 — Description of Additional Supplementary Files [file 41467_2022_28121_MOESM2_ESM.pdf]

Description of Additional Supplementary Files

File Name: Supplementary Data 1
Description: *Shigella* and *E. coli*, strains isolates and genomes used in this study. The following are shown: name, collection year, country of isolation, species, *Shigella* serotype, cluster, dataset, MLST7 type, cgMLST HC2350 type, cgMLST HC2000 type, cgMLST HC1100 type, cgMLST HC400 type, EnteroBase barcode, GenBank and EBI-ENA accession numbers, BioProject number, reference, genome coverage, N50, genome length, no. of contigs.
