# Supplementary material for: Population structure analysis and laboratory monitoring of Shigella by core-genome multilocus sequence typing
Source: Nat Commun. 2022 Jan 27;13:551. doi: 10.1038/s41467-022-28121-1 (PMC8795385; doi:10.1038/s41467-022-28121-1)
Supplement: Supplementary file 4 — Reporting Summary [file 41467_2022_28121_MOESM4_ESM.pdf]

nature research

Corresponding author(s): François-Xavier WEILL

Last updated by author(s): Dec 11, 2021

Reporting Summary

Nature Research wishes to improve the reproducibility of the work that we publish. This form provides structure for consistency and transparency in reporting. For further information on Nature Research policies, see our Editorial Policies and the Editorial Policy Checklist.

Statistics

For all statistical analyses, confirm that the following items are present in the figure legend, table legend, main text, or Methods section.

n/a	Confirmed	
☐	☒	The exact sample size (*n*) for each experimental group/condition, given as a discrete number and unit of measurement
☒	☐	A statement on whether measurements were taken from distinct samples or whether the same sample was measured repeatedly
☒	☐	The statistical test(s) used AND whether they are one- or two-sided<br>*Only common tests should be described solely by name; describe more complex techniques in the Methods section.*
☒	☐	A description of all covariates tested
☒	☐	A description of any assumptions or corrections, such as tests of normality and adjustment for multiple comparisons
☒	☐	A full description of the statistical parameters including central tendency (e.g. means) or other basic estimates (e.g. regression coefficient) AND variation (e.g. standard deviation) or associated estimates of uncertainty (e.g. confidence intervals)
☒	☐	For null hypothesis testing, the test statistic (e.g. *F*, *t*, *r*) with confidence intervals, effect sizes, degrees of freedom and *P* value noted<br>*Give P values as exact values whenever suitable.*
☒	☐	For Bayesian analysis, information on the choice of priors and Markov chain Monte Carlo settings
☒	☐	For hierarchical and complex designs, identification of the appropriate level for tests and full reporting of outcomes
☒	☐	Estimates of effect sizes (e.g. Cohen's *d*, Pearson's *r*), indicating how they were calculated

Our web collection on statistics for biologists contains articles on many of the points above.

Software and code

Policy information about availability of computer code

Data collection	Excel version 15.41
Data analysis	FqCleanER version 3.0 (https://gitlab.pasteur.fr/GIPhy/fqCleanER), NCBI BLASTn blast+/v.2.10.1 (https://blast.ncbi.nlm.nih.gov/Blast.cgi), ORFfinder (https://www.ncbi.nlm.nih.gov/orffinder/), EnteroBase (http://enterobase.warwick.ac.uk/), SeroPred (http://enterobase.warwick.ac.uk/), ShigaTyper (https://github.com/CFSAN-Biostatistics/shigatyper), ShigEiFinder (https://github.com/LanLab/ShigEiFinder), GrapeTree (http://enterobase.warwick.ac.uk/), Snippy v4.6 (https://github.com/tseemann/snippy), SPAdes version 3.15 (https://cab.spbu.ru/software/spades/), RAxML-NG v1.0.1 (https://github.com/amkozlov/raxml-ng), iTOL version 6 (https://itol.embl.de), MEGA X v.10.2.1 (https://www.megasoftware.net/),pHierCC (http://enterobase.warwick.ac.uk/), Gubbins version 2.4.1 (https://github.com/sanger-pathogens/gubbins).

For manuscripts utilizing custom algorithms or software that are central to the research but not yet described in published literature, software must be made available to editors and reviewers. We strongly encourage code deposition in a community repository (e.g. GitHub). See the Nature Research guidelines for submitting code & software for further information.

Data

Policy information about availability of data

All manuscripts must include a data availability statement. This statement should provide the following information, where applicable:

- Accession codes, unique identifiers, or web links for publicly available datasets
- A list of figures that have associated raw data
- A description of any restrictions on data availability

Short-read sequence data were submitted to EnteroBase (https://enterobase.warwick.ac.uk/) and to the European Nucleotide Archive (ENA, https://www.ebi.ac.uk/ena/) under study numbers PRJEB44801, PRJEB2846, and PRJEB2128. Other whole genome sequences analysed during the study are available from ENA (https://

www.ebi.ac.uk/ena/), NCBI RefSeq (https://www.ncbi.nlm.nih.gov/refseq/), DDBJ (https://www.ddbj.nig.ac.jp/index-e.html), and GenBank (https://www.ncbi.nlm.nih.gov/genbank/). All the accession numbers of the genomes used in this study are listed in Supplementary Data 1. The GrapeTree of 493 Shigella and E. coli reference genomes is publicly available from EnteroBase (http://enterobase.warwick.ac.uk/ms_tree?tree_id=55118) and from Microreact (https://microreact.org/project/kP4HJjriDvAfTS4Ed3Avx8/01568b6f). The nucleotide sequences of the Shigella rfb clusters were submitted to GenBank (https://www.ncbi.nlm.nih.gov/genbank/) under accession numbers MZ286364-MZ28639, MZ303046, MF322747-MF322752, and MF322754. The accession numbers for the individual rfb sequences are given in Supplementary Table 2. All custom scripts can be found at https://github.com/imanyass/Shigella_population_2021

Field-specific reporting

Please select the one below that is the best fit for your research. If you are not sure, read the appropriate sections before making your selection.

☒ Life sciences ☐ Behavioural & social sciences ☐ Ecological, evolutionary & environmental sciences

For a reference copy of the document with all sections, see nature.com/documents/nr-reporting-summary-flat.pdf

Life sciences study design

All studies must disclose on these points even when the disclosure is negative.

Sample size	No calculations were performed to determine sample size. We have sequenced 306 Shigella reference strains (average of 5 reference strains per serotype) and 3870/3942 (98.2%) clinical isolates (2017-2020) available at the French National Reference Center for Escherichia coli, Shigella and Salmonella, Institut Pasteur, Paris, France (n = 4,176). We have also included 11 Shigella strains belonging to provisional Shigella serotypes and provided by international reference laboratories. We also selected 81 additional Shigella genomes in EnteroBase for new HC1100/HC400/in silico serotype combinations. Finally, 95 E. coli genomes, including 27 Entero invasive E. coli (EIEC) from eight different EIEC genomic clusters and 68 (of the 72) strains from the ECOR collection, were also included to place our Shigella genomes in the phylogenetic context of the broader diversity of E. coli. No statistical analysis was carried out.
Data exclusions	One Shigella boydii 7 (UE 95-1589) and all Shigella boydii 13 were excluded from this study because they belonged to another species, Escherichia albertii. Seventy-two clinical isolates were excluded because (i) they were redundant (two or more isolates from the same person in less than two months) and consequently not sequenced (ii) or their genomic sequences were contaminated with another bacterial species. Four E. coli ECOR strains (ECOR 7, 23, 32, 43) were also excluded due to discrepant results for MLST and/or Clermont typing between the studies of Galardini et al, Clermont et al, and the data from EnteroBase.
Replication	All experiments were successfully replicated at least twice.
Randomization	Not applicable, this study does not involve human participants or animal models.
Blinding	Not applicable, this study is not a clinical trial

Reporting for specific materials, systems and methods

We require information from authors about some types of materials, experimental systems and methods used in many studies. Here, indicate whether each material, system or method listed is relevant to your study. If you are not sure if a list item applies to your research, read the appropriate section before selecting a response.

Materials & experimental systems

n/a	Involved in the study	
☒	☐	Antibodies
☒	☐	Eukaryotic cell lines
☒	☐	Palaeontology and archaeology
☒	☐	Animals and other organisms
☒	☐	Human research participants
☒	☐	Clinical data
☒	☐	Dual use research of concern

Methods

n/a	Involved in the study	
☒	☐	ChIP-seq
☒	☐	Flow cytometry
☒	☐	MRI-based neuroimaging
